# Genome-scale analysis and comparison of gene expression profiles in developing and germinated pollen in *Oryza sativa*

**DOI:** 10.1186/1471-2164-11-338

**Published:** 2010-05-28

**Authors:** Li Q Wei, Wen Y Xu, Zhu Y Deng, Zhen Su, Yongbiao Xue, Tai Wang

**Affiliations:** 1 Research Center of Molecular and Developmental Biology, Key Laboratory of Photosynthesis and Environmental Molecular Physiology, Institute of Botany, Chinese Academy of Sciences, Beijing 100093, China; 2 National Center for Plant Gene Research, Beijing 100093, China; 3 Key Laboratory of Molecular and Developmental Biology, Institute of Genetics and Developmental Biology, Chinese Academy of Sciences, and National Center for Plant Gene Research, Beijing 10010, China; 4 Division of Bioinformatics, State Key Laboratory of Plant Physiology and Biochemistry, College of Biological Sciences, China Agricultural University, Beijing 100094, China

## Abstract

**Background:**

Pollen development from the microspore involves a series of coordinated cellular events, and the resulting mature pollen has a specialized function to quickly germinate, produce a polar-growth pollen tube derived from the vegetative cell, and deliver two sperm cells into the embryo sac for double fertilization. The gene expression profiles of developing and germinated pollen have been characterised by use of the eudicot model plant *Arabidopsis*. Rice, one of the most important cereal crops, has been used as an excellent monocot model. A comprehensive analysis of transcriptome profiles of developing and germinated pollen in rice is important to understand the conserved and diverse mechanism underlying pollen development and germination in eudicots and monocots.

**Results:**

We used Affymetrix GeneChip^® ^Rice Genome Array to comprehensively analyzed the dynamic changes in the transcriptomes of rice pollen at five sequential developmental stages from microspores to germinated pollen. Among the 51,279 transcripts on the array, we found 25,062 pollen-preferential transcripts, among which 2,203 were development stage-enriched. The diversity of transcripts decreased greatly from microspores to mature and germinated pollen, whereas the number of stage-enriched transcripts displayed a "U-type" change, with the lowest at the bicellular pollen stage; and a transition of overrepresented stage-enriched transcript groups associated with different functional categories, which indicates a shift in gene expression program at the bicellular pollen stage. About 54% of the now-annotated rice F-box protein genes were expressed preferentially in pollen. The transcriptome profile of germinated pollen was significantly and positively correlated with that of mature pollen. Analysis of expression profiles and coexpressed features of the pollen-preferential transcripts related to cell cycle, transcription, the ubiquitin/26S proteasome system, phytohormone signalling, the kinase system and defense/stress response revealed five expression patterns, which are compatible with changes in major cellular events during pollen development and germination. A comparison of pollen transcriptomes between rice and *Arabidopsis *revealed that 56.6% of the rice pollen preferential genes had homologs in *Arabidopsis *genome, but 63.4% of these homologs were expressed, with a small proportion being expressed preferentially, in *Arabidopsis *pollen. Rice and *Arabidopsis *pollen had non-conservative transcription factors each.

**Conclusions:**

Our results demonstrated that rice pollen expressed a set of reduced but specific transcripts in comparison with vegetative tissues, and the number of stage-enriched transcripts displayed a "U-type" change during pollen development, with the lowest at the bicellular pollen stage. These features are conserved in rice and *Arabidopsis*. The shift in gene expression program at the bicellular pollen stage may be important to the transition from earlier cell division to later pollen maturity. Pollen at maturity pre-synthesized transcripts needed for germination and early pollen tube growth. The transcription regulation associated with pollen development would have divergence between the two species. Our results also provide novel insights into the molecular program and key components of the regulatory network regulating pollen development and germination.

## Background

The formation of highly specialized haploid male gametophytes (pollen) from microspores involves a series of cellular events. The microspore released from tetrads quickly increases in size and then undergoes asymmetric mitosis (pollen mitosis I [PMI]) to generate a large vegetative cell and a small generative cell. Thereafter, the generative cell undergoes second mitosis (PMII), giving rise to two sperm cells, and the vegetative cell exits the cell cycle[1]. As a result, the pollen is specialized in function, and during pollination, it can quickly germinate, produce a vegetative cell-derived polarly growing pollen tube, and deliver the two sperm cells into the embryo sac to initiate double fertilization. In addition to its intrinsic function for sexual reproduction, pollen represents an excellent model system for uncovering the molecular mechanisms of fundamental cellular processes such as cell division, differentiation, fate determination, polar establishment, cell-to-cell recognition and communication [1,2].

Molecular genetic and biochemical studies have identified several genes involved in the regulation of pollen development, specifically those in germ division and sperm specification mainly in *Arabidopsis *[1,3-5]. For example, *TIO *and *kinesin-12A/kinesin-12B *are required for PMI, and *DUO1, DUO3, CDKA;1 *and *FBL 17 *for PMII [1,6]. A recent study showed that *DUO1*, a germline-specific R2R3 Myb gene, is a key regulator integrating cell cycle progression and sperm specification [7]. However, compared with knowledge of the complicated and ordered cellular events leading to pollen maturation, which are accompanied by serial changes in gene expression profiles [1,4,5,8], our knowledge about the mechanism underlying pollen development is still limited.

Several studies have analyzed transcriptome features of mature pollen from *Arabidopsis *by 8 K Affymetrix AG microarrays (representing about 30% genes of the genome) [9,10] or GeneChip ATH1 arrays (representing about 80% genes of the genome)[11]. These studies revealed that mature pollen has a smaller and more unique transcriptome with a high proportion of selectively expressed genes than do vegetative tissues. These genes expressed selectively in pollen are functionally skewed towards cell wall metabolism, signalling and cytoskeleton dynamics [9-11], which suggests that the transcriptional characteristics involve pollen function specialization. Lee and Lee [12] compared gene expression profiles of *Arabidopsis *pollen under normal and cold-stress conditions using serial analysis of gene expression technology and found that most of the genes expressed in pollen were not affected by cold stress. Haerizadeh et al. [13] identified transcripts expressed selectively in mature pollen of soybean (*Glycine max*) and revealed that the transcriptome had a high proportion of transcripts involved in signalling, transcription, heat shock response, transporting and the ubiquitin/proteasome pathway. Importantly, Honys and Twell [14] systematically analyzed dynamic transcriptome profiles of pollen development from microspore to mature stages in *Arabidopsis *using GeneChip ATH1 arrays and revealed a decrease in proportion of transcript species and an increase in the proportion of pollen-specific transcripts during the development process. By using the same *Arabidopsis *ATH1 array, Borges et al [15] revealed most of these sperm cell-expressed genes were also expressed in mature pollen and 11% of them were enriched expression in the sperm cell. These sperm cell-enriched transcripts were preferentially involved in DNA repair, ubiquitin-mediated proteolysis and cell cycle progression. Additionally, a comparison of the pollen transcriptome of mutants deficient in different MIKC* protein complexes and wild type plants in *Arabidopsis *showed the absence of the protein complexes affected the expression of more than 1300 genes during pollen maturation [16]. These studies gave a comprehensive picture of temporal dynamics of gene expression profiles in pollen development.

Pollen germination and polar tube growth need a coordinated action of multiple cellular and biochemical events; and the tip-focused intracellular Ca^2+ ^gradient and tip plasma membrane-localized Rop1 GTPase have been well documented as being important factors for regulation of polar establishment and growth of pollen tubes [17]. In contrast to the increased molecular information about development and maturity of pollen, that about genome-wide events underlying pollen germination and tube growth, which are essential for understanding the molecular mechanisms of polar tube growth and invasion into pistils, is limited. Inhibition experiments with numerous plant species including *Arabidopsis*, demonstrate that pollen germination and polar tube growth strictly depend on protein synthesis and are relatively independent of transcription [14,18-20]. In *Arabidopsis*, relative to mature pollen, hydrated pollen and pollen tubes have a large number of newly transcribed genes [21]. Thus, *de novo *synthesis of transcripts in germinating pollen may be not crucial to germination and early tube growth, although the issue needs to be addressed by sequential comparison of transcriptomes from developing and germinated pollen.

Rice, one of the most important cereal crops, is the staple food for half of the world's population and has been used as an excellent model after *Arabidopsis*, because of its relatively smaller genome and the completion of the genome sequence. Rice pollen has several features different from *Arabidopsis *pollen. Rice pollen is representative of wind-pollinated tricellular pollen and has a thinner wall (0.8~1.2 μm) and fewer lipids in the coat layer than do other plants in Gramineae [22,23]. In addition, rice pollen appears to have less longevity under *in vitro *conditions than does dicot pollen [23] and loses germination activity in a short period of time [23-25]. Russell et al. [26] identified putative pollen allergens and gene organization of these allergens in rice on a genome-wide scale. To help understand the molecular regulation of pollen development and germination, we analyzed transcriptome features of rice pollen during five sequential developmental stages, from microspores to germinated pollen, using the Affymetrix GeneChip^® ^Rice Genome Array. These results shed light on the overall characteristics of expression profiles associated with pollen development and germination and on the molecular program and key components of the regulatory network regulating the processes.

## Results

### Isolation and characterization of developing and *in vitro*-germinated pollen

**Figure 1:**
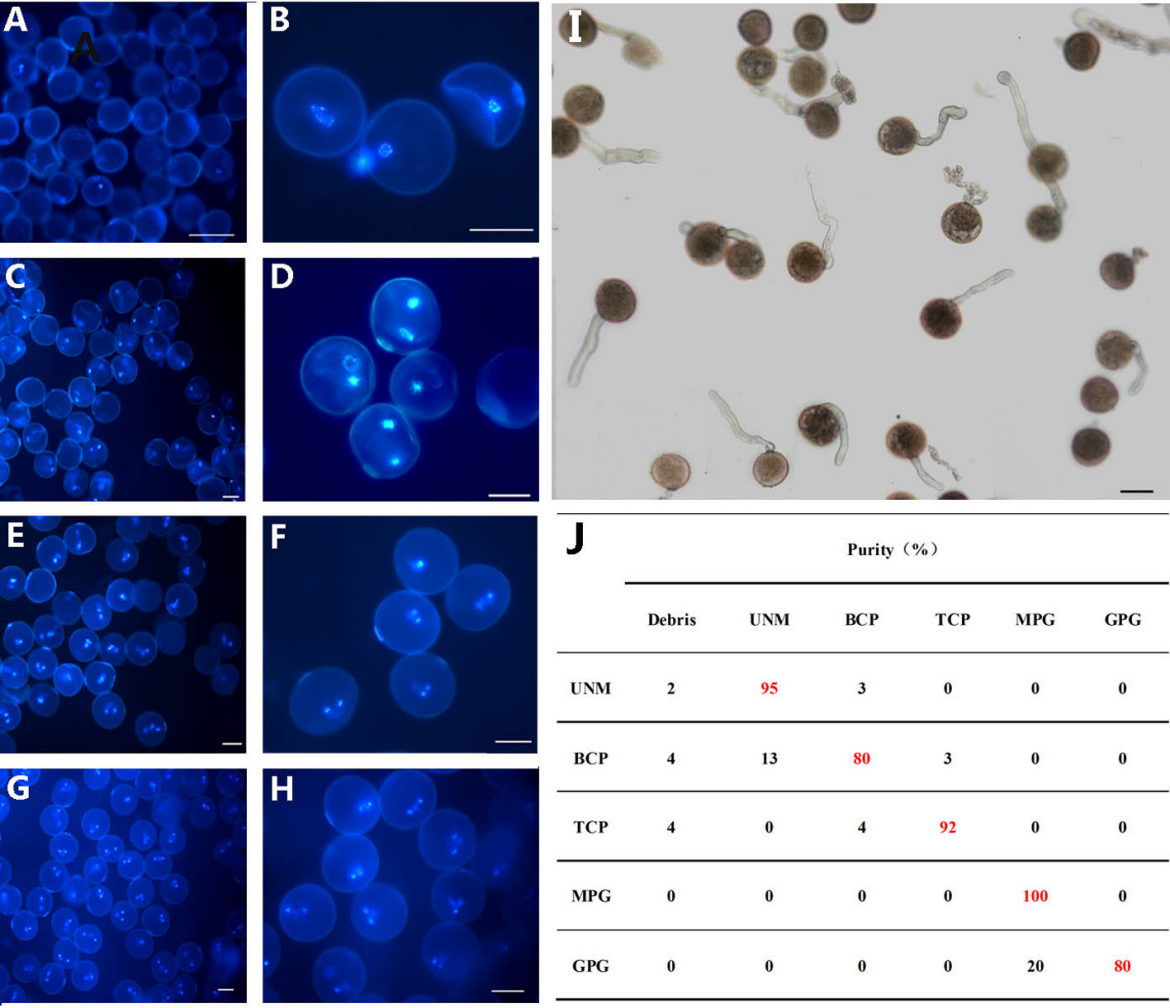
**Characterization of the isolated and purified pollen at respective stage**. **(A-H) **DAPI staining of the purified pollen: **(A, B) **Uninucleate microspores (UNMs). **(C, D) **Bicellular pollen (BCP). **(E, F) **Tricellular pollen (TCP). **(G, H) **Mature pollen grains (MPGs). **(I) **Germinated pollen grains (GPGs). **(J) **The purity of the purified pollen was determined by DAPI staining and microscopy, and percentage of GPGs. Bars = 100 μm in A to I.

To analyze changes in expression profiles during pollen development and germination, we isolated and purified uninucleate microspores (UNMs), bicellular pollen (BCP) and immature tricellular pollen (TCP) (see Materials and Methods) with purity of about 95%, 80% and 92%, respectively (Figure 1A~F, and 1J). Mature pollen grains (MPGs) were collected from blossoming flowers, and all were tricellular (Figure 1G~H). On fluorescin 3', 6'-diacetate staining, more than 90% of the isolated UNMs, BCP, TCP and MPGs were viable (data not shown). The condition used for pollen germination resulted in germinated pollen grains (GPGs) of 80% (Figure 1I and 1J).

### Transcriptome characteristics in developing and germinated pollen

**Figure 2:**
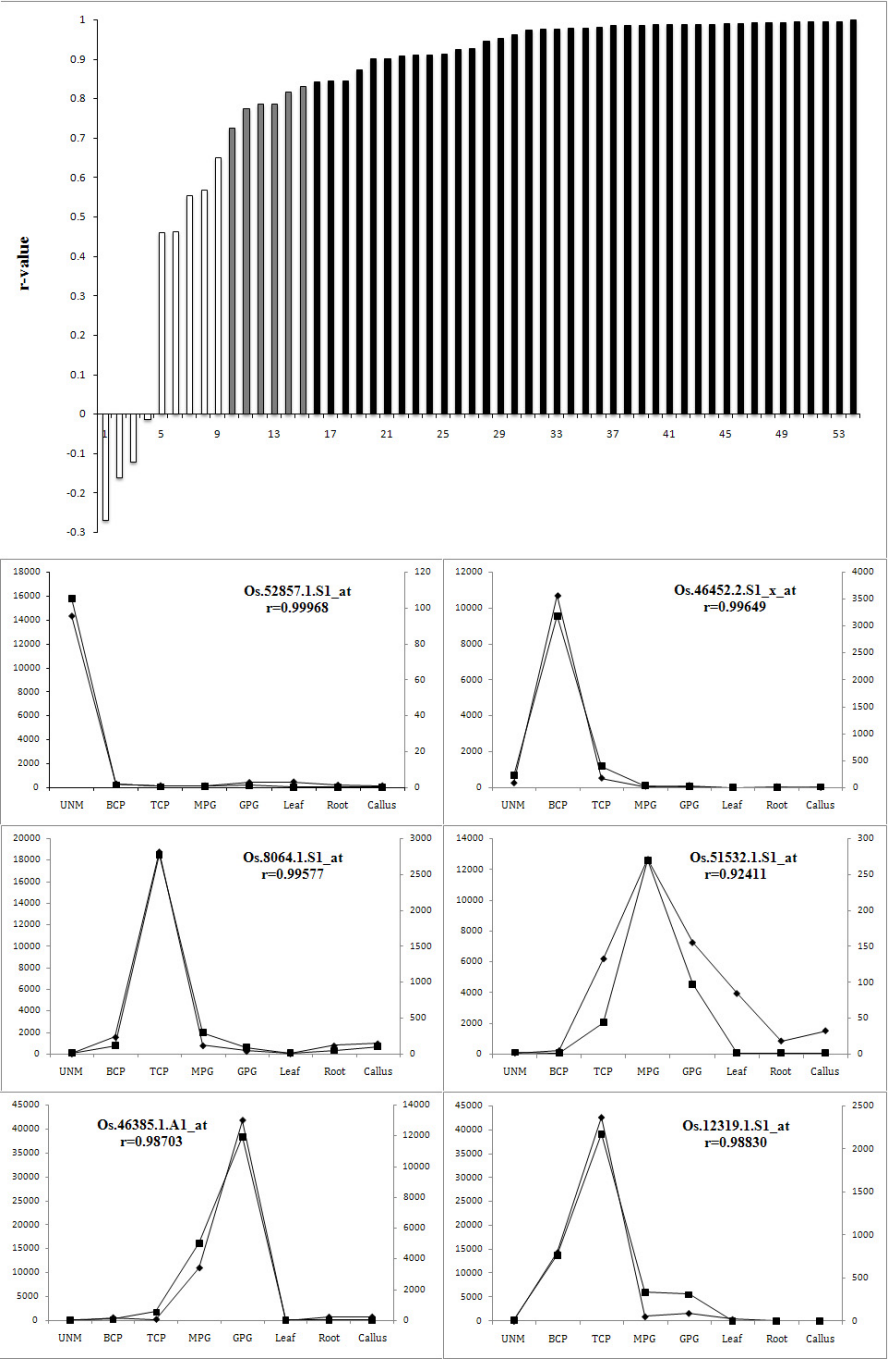
**Confirmation of expression profiles of 54 transcripts by quantitative RT-PCR (qRT-PCR)**. The column shows the correlation between two types of expression profiles of the 54 transcripts from GeneChip^® ^and qRT-PCR data. Among the 54 transcripts, the correlation coefficients (r-value) between the two type of expression profiles were more than 0.707 (P < 0.05) for 45 transcripts (grey and black columns), and for 40/45 transcripts, the r-values were more than 0.834 (P < 0.01, black columns) (the numbers in the X-axis represented the serial number (SN) of the corresponding transcripts in Additional file 1, and the detailed data in Additional file 1). Six examples of the two profile types are shown below the column, and the y-axis is the expression level from GeneChip^® ^(left) and qRT-PCR (right) (, expression profiles detected by GeneChip; , expression profiles by qRT-PCR).

We analyzed genome-wide gene expression profiles of all five samples -- UNMs, BCP, TCP, MPGs and GPGs -- along with sporophytic tissue callus cells, roots and leaves as controls, using the Affymetrix GeneChip^® ^Rice Genome Array. Three independent biological replicates were performed for each sample. The correlation coefficient value for each experiment was larger than 0.93. Real-time quantitative RT-PCR (qRT-PCR) analysis was used to confirm the array data. In total, 54 pollen-preferential/stage-enriched transcripts were analyzed (Additional file 1). The signal intensity values of these examined transcripts ranged from 189,609 to 1.1, and the ratio was from 22,992.57 to 4.71 (Additional file 1). The profiles produced by qRT-PCR and GeneChip^® ^Rice Genome Array showed a significant positive correlation for 45 of the 54 genes (r > 0.707, P < 0.05) (Figure 2), which demonstrated that about 83% of the microarray expression data could be confirmed by qRT-PCR.

The GeneChip^® ^Rice Genome Array contains probes to query 51,279 transcripts http://www.affymetrix.com. Analyses involving the microarray suite (MAS) 5.0 detection algorithm revealed 14,590 genes expressed in UNMs, 12,967 in BCP, 12,514 in TCP, 5939 in MPGs and 5945 in GPGs, in comparison with 16,000 in callus cells, 17,383 in roots and 17,424 in leaves (Additional file 2). To be cautious, we re-analyzed the raw data using DNA-Chip Analyzer (dChip) and obtained the same results. Thus, the developing and germinated pollen have a smaller transcriptome than do sporophytic tissues. These data also show greatly decreased transcript diversity from UNMs to MPGs, similar to the observation in *Arabidopsis *pollen [14]; whereas the transcript diversity of GPGs was similar to that of MPGs.

Furthermore, we analysed the correlation among the transcriptome profiles of pollen at different stages (Additional file 3). Because callus cells have active cell division activity and are a mass of undifferentiated cells, the transcriptome profile of the callus cells was used as a reference. With advanced pollen development, the similarities between the pollen and callus cell transcriptome profiles decreased greatly, from the highest for UNMs (r = 0.72) to the lowest for MPGs (r = 0.11). Accordingly, the expression profile for UNMs was more similar to that for BCP (r = 0.82) than to that for other stages, with the lowest similarity to the transcriptome profiles for MPGs and GPGs (r = 0.13). A similar result was also observed in *Arabidopsis *[14]. The findings suggested that UNMs appeared to use an expression program similar to that of callus cells for cell proliferation, and thereafter, the program in developing pollen was specified for pollen functional specialization. Interestingly, in line with the observation of MPGs and GPGs having almost the same number of diverse transcripts, the gene expression profiles of MPGs and GPGs were significantly and positively correlated (r = 0.99), which suggests that MPGs have stored a set of transcripts that will be used for germination and early tube growth. Combined with results of several previous studies of inhibiting transcription and translation which have showed pollen germination and early tube growth strictly depend on protein synthesis but not transcription [14,18-20], our results confirmed transcript storage through pollen germination in rice.

### Identification of development stage-enriched and -downregulated genes

We identified development stage-enriched genes by comparing the expression levels of genes in developing and germinated pollen and in sporophytic tissues (callus cells, roots and leaves) using the Z-score transformation normalization method [27], with cutoffs of ratio ≥ 2.0 and Z-score ≥ 3.75. The analysis revealed 2,203 development stage-enriched probe sets: 660 (corresponding to 568 unigenes) expressed preferentially in UNMs, 174 (146 unigenes) in BCP, 246 (198 unigenes) in TCP, 537 (296 unigenes) in MPGs and 586 (358 unigenes) in GPGs (Additional file 4b1, c1, d1, e1 and 4f1). This finding indicated a "U-type" change tendency in number of stage-enriched transcripts during the process, and GPGs appeared to have a number of enriched transcripts similar to that of MPGs. Furthermore, we used the same method to screen stage-enriched genes in *Arabidopsis *developing pollen from an available database [14] and found 190 genes expressed preferentially in UNMs, 94 in BCP, 161 in TCP, and 313 in MPGs. These data indicated that in both rice and *Arabidopsis*, the number of stage-enriched transcripts decreased sharply from UNMs to BCP and thereafter increased to a maximum level in MPGs. Relative to stage-enriched genes, genes downregulated in a development stage showed a distinct feature. Using the same parameter cut-offs of ratio ≤ 0.5 and Z-score ≤ -3.75 as for screening stage-enriched genes, we did almost not identified transcripts downregulated in each stage (only 1 in UNMs, 1 in BCP, none in TCP, 1 in MPGs and 3 in GPGs). Therefore, we used relatively low stringent parameters (ratio ≤ 0.5 and Z-score <-1.7245 (p-value < 0.05)) to screen genes downregulated at each stage. The analysis revealed that 70 transcripts (corresponding to 65 unigenes) downregulated in UNMs, 52 (49 unigenes) in BCP, 143 (138 unigenes) in TCP, 429(411 unigenes) in MPGs, and 530 (507 unigenes) in GPGs (Additional file 4b2, c2, d2, e2 and 4f2), which suggested that stage-downregulated genes were substantially increased in number after the bicellular pollen stage.

**Figure 3:**
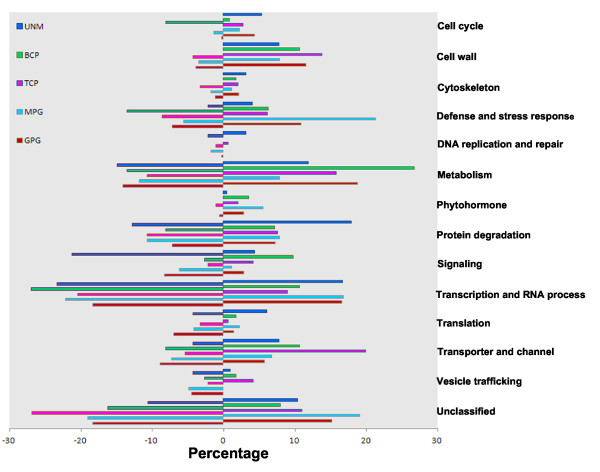
**Distribution of functionally annotated development stage-enriched and -downregulated genes in the different functional groups**. The genes were classified into 13 functional groups and one unclassified group. Positive and negative numbers denote the percentage of stage-enriched and -downregulated functional groups, respectively. Corresponding detailed information is listed in Additional file 5a and b (stage-enriched) and c and d (stage-downregulated).

Furthermore, we analyzed the functional features of these transcripts according to a part and/or an instance of the parent of gene ontology (GO) terms and BLAST search results of transcripts without GO terms. Not taking into account the unknown transcripts (annotated as "expressed" or "hypothetical" or with no annotated coding sequence information in the TIGR rice genome annotation database http://rice.plantbiology.msu.edu/) and the transposable element-related transcripts, the remaining development stage-enriched and -downregulated genes could be classified into 13 functional groups and one "unclassified" group in which the genes had putative functional information but could not be classified clearly into the above 13 groups (Figure 3). The distribution of stage-enriched and -downregulated genes showed obviously dynamic change at distinct stages (Figure 3 and Additional file 5). Both stage-enriched and -downregulated transcripts showed a functional skew toward metabolism, transcription/RNA process, and protein degradation; the latter two groups are important in regulation of transcription and protein turnover. This finding suggested that a network of transcription and protein turnover regulation is required for pollen development and function specification. However, functional features of the two datasets were strikingly different in below aspects. First, relative to stage-enriched genes associated with the cell cycle, cytoskeleton, cell wall and phytohormones, stage-downregulated genes implicated in these functional terms were in much lower number and were obviously stage-dependent. For example, stage-downregulated cell cycle-related transcripts preferentially occurred in BCP, and fewer phytochormone-related transcripts were identified in the stage-downregulated dataset. Second, among the stage-downregulated genes, signalling-related transcripts were overrepresented in UNMs, but among stage-enriched genes, such transcripts were overrepresented in BCP. Third, the number of defense/stress response-related transcripts increased greatly from UNMs and peaked in MPGs among the stage-enriched dataset, whereas decreased from BCP in the stage down regulated dataset. Unexpectedly, stage-downregulated transcripts implicated in vesicle trafficking were identified in pollen of all stages, but stage-enriched transcripts implicated in the functional term were only in pollen from UNM to TCP stages. This suggested that development stage-dependent enrichment or downregulation of different genes is required for pollen development and function specification.

**Figure 4:**
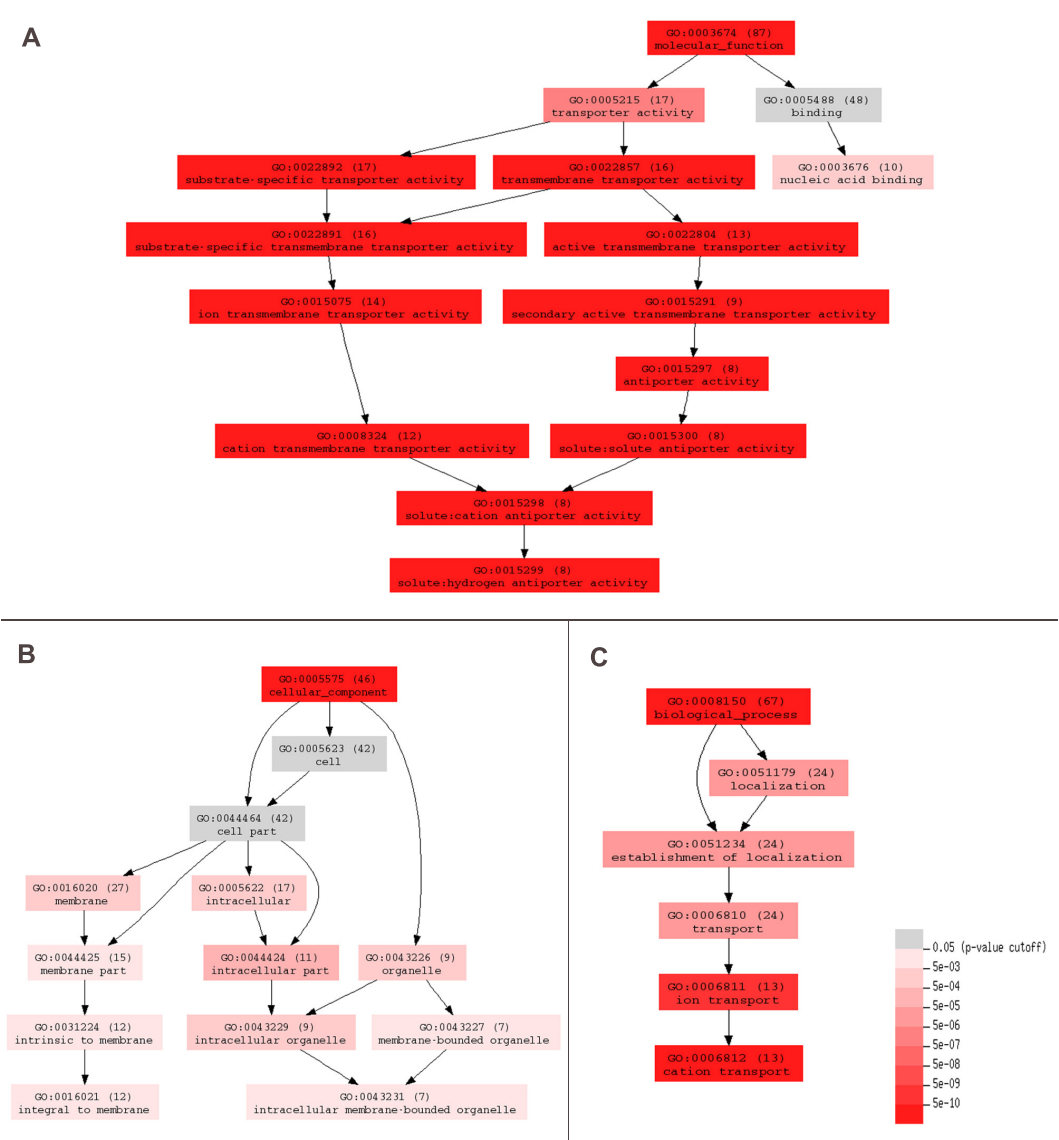
**Gene ontology (GO) term "enrichment status" for the development stage-enriched transcripts in TCP**. Transcript with GO term "enrichment status" and "hierarchy" in A), "molecular function"; B), "cellular component"; and C), "biological process" branches. The classification terms and their serial numbers are represented as rectangles. The numbers in brackets represent the total number of genes that may be involved in the corresponding biological processes. The graph displays the classification terms "enrichment status" and "hierarchy". The color scale shows the *p-*value cutoff levels for each biological process. The deeper colors represent the more significant biological processes in the putative pollen pathway.

Analysis of development stage-enriched transcripts in the molecular functional and cellular component terms "enrichment status" and "hierarchy" showed that cation transmembrane transporter and antiporter activity had statistical importance in TCP (Figure 4A and 4C). Transcripts involved in membrane and membrane-bounded organelle seemed to be more important in TCP-enriched data (Figure 4B). Vesicle trafficking activity was statistically significant in MPGs and GPGs in the development stage-downregulated dataset (Additional file 6).

### Identification of genes expressed preferentially in developing and germinated pollen

**Figure 5:**
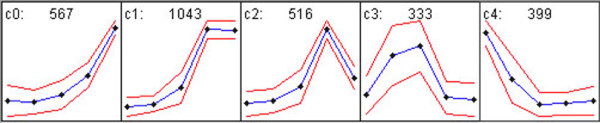
**Expression patterns analysis of 2,858 pollen-preferential transcripts of six functional groups**. The 2,858 transcripts, involving transcription factors, phytohormones, kinases, cell cycle regulators, ubiquitin/26 S proteasome system and defence/stress responders, were distributed in five clusters (c0 to c4). The clusters were created by GeneCluster 2.0; raw data for each cluster are listed in Additional file 7. X-axis denotes the developmental stages from UNM to GPG; y-axis denotes the normalized expression level of the transcripts.

To extensively analyze the molecular regulation of pollen development and germination, we further screened transcripts expressed preferentially in pollen (one or more stage) using sporophytic tissues as controls (ratio ≥ 2.0, Z-score ≥ 3.19) and those shared across all pollen stages and sporophytic tissues (see Materials and Methods). The screening identified 25,062 pollen-preferential transcripts (Additional file 4a) and 10,777 transcripts expressed constitutively both in pollen and sporophytic tissues (Additional file 4h). Relative to the constitutively expressed transcripts that may have a housekeeping function in pollen and sporophytic development, the pollen-preferential transcripts have an important function in pollen development. Therefore, we focused on the expression characteristics of 6 functional groups (2,858 of 25,062 preferentially expressed transcripts) related to cell cycle regulation, phytohormone signalling, the kinase system, the ubiquitin/26 S proteasome system (UPS), transcription, and defense/stress response that have diversely important roles in different tissues, including pollen, as revealed by numerous studies (see below sections). Cluster analysis of the 2,858 transcripts revealed 5 expression patterns (Figure 5 and Table 1). The largest cluster was cluster 1 (c1), with 1,043 transcripts whose expression was at the lowest level in UNMs and thereafter increased to the highest level in MPGs and GPGs. The second and third largest clusters were clusters 0 (c0; 567) and 2 (c2; 516), respectively. Transcripts in c0 were up-regulated from UNMs to GPGs. The expression level of transcripts in c2 increased from UNMs to MPGs but decreased from MPGs to GPGs. Cluster 3 (c3), of 333 transcripts, began to be up-regulated in UNMs, peaked at BCP and TCP, and decreased thereafter. Cluster 4 (c4) consisted of 399 transcripts, whose expression was highest in UNMs, was reduced to a relatively low level in TCP and remained at a low level in the following stages. Transcripts involved in distinct functional groups showed heterogeneous distribution in the 5 clusters. For example, most transcripts of calcium signal-related kinases were in c1 (19/33), which is in line with known important roles of calcium signalling in germination and polar tube growth [28]. Therefore, the change in expression patterns of distinct transcripts suggests the requirement of different development events from UNMs to GPGs.

**Table 1:** Distributions of six functional-group pollen-preferential transcripts in the five clusters.

Categories/sub-categories	c0	c1	c2	c3	c4	Total
**01 Trancription factor**	**176**	**282**	**133**	**89**	**92**	**772**
01.01 ABI3VP1	4	8	6	4	2	24
01.02 Alfin-like	1	1	0	2	1	5
01.03 AP2-EREBP	18	25	11	4	5	63
01.04 ARF	1	1	2	1	3	8
01.05 ARID	1	1	0	0	0	2
01.06 ARR-B	0	1	0	0	0	1
01.07 BES1	0	2	1	0	0	3
01.08 bHLH	11	23	10	5	2	51
01.09 bZIP	9	9	5	2	0	25
01.10 C2C2-CO-like	1	0	0	0	1	2
01.11 C2C2-Dof	5	7	0	0	0	12
01.12 C2C2-GATA	2	5	0	1	2	10
01.13 C2C2-YABBY	1	1	0	1	0	3
01.14 C2H2	12	18	9	6	9	54
01.15 C3 H	4	11	1	2	9	27
01.16 C3HC4	1	0	1	3	1	6
01.17 CCAAT_HAP2	0	2	1	0	0	3
01.18 CCAAT_HAP3	1	4	0	1	0	6
01.19 CCAAT_HAP5	2	2	0	3	2	9
01.20 CPP	1	2	1	0	1	5
01.21 CSD	0	0	0	0	1	1
01.22 DBP	0	3	0	1	0	4
01.23 DDT	3	0	3	0	1	7
01.24 G2-like	3	3	2	1	2	11
01.25 GeBP	1	0	0	1	0	2
01.26 GRAS	6	6	6	2	0	20
01.27 GRF	0	1	0	0	0	1
01.28 HB	7	15	5	5	0	32
01.29 HMG	0	1	1	0	2	4
01.30 HRT	0	1	0	0	0	1
01.31 HSF	2	1	1	1	1	6
01.32 Jumonji	3	2	1	2	0	8
01.33 LFY	0	1	0	0	0	1
01.34 LIM	0	3	0	0	0	3
01.35 LUG	0	0	1	0	0	1
01.36 MADS	10	13	8	5	8	44
01.37 MYB and MYB-related	20	26	18	7	12	83
01.38 NAC	9	26	8	2	6	51
01.39 Orphans	14	17	10	2	2	45
01.40 PHD	4	5	3	4	2	18
01.41 PLATZ	1	0	1	1	0	3
01.42 Pseudo ARR-B	0	1	0	0	0	1
01.43 PWP-RK	1	4	0	0	0	5
01.44 SBP	1	1	2	0	0	4
01.45 SET	3	2	2	6	7	20
01.46 S1Fa-like	0	0	0	2	0	2
01.47 SNF2	4	3	2	3	6	18
01.48 TAZ	0	0	0	0	1	1
01.49 TCP	3	3	2	0	2	10
01.50 Trihelix	0	4	1	0	0	5
01.51 TUB	1	1	1	3	0	6
01.52 ULT	0	0	1	1	0	2
01.53 WRKY	3	9	3	4	1	20
01.54 zf-HD	2	3	2	0	0	7
01.55 ZIM	0	4	1	1	0	6
**02 Phytohormone**	**31**	**52**	**28**	**25**	**10**	**146**
02.01 Auxin	13	31	16	13	7	80
02.02 BR	3	2	2	2	1	10
02.03 Cytokinin	2	3	3	0	0	8
02.04 Ethylene	6	13	3	3	2	27
02.05 GA	3	2	0	6	0	11
02.06 JA	4	1	4	1	0	10
**03 Cell cycle**	**36**	**63**	**30**	**25**	**43**	**197**
**04 Kinase**	**110**	**319**	**116**	**78**	**43**	**666**
04.01 Calcium	4	19	2	6	2	33
04.02 Inositol phosphate	6	21	7	5	0	39
04.03 MAPK	1	11	1	3	0	16
04.04 Pto-pti	1	5	0	1	0	7
04.05 Receptor kinase	46	83	43	20	5	197
04.06 WAK	10	14	10	1	2	37
04.07 Others	42	166	53	42	34	337
**05 Defense/stress response**	**107**	**172**	**109**	**51**	**35**	**474**
**06 UPS**	**107**	**155**	**100**	**65**	**176**	**603**
06.01 F-box	75	103	71	49	108	406
06.02 Others	32	52	29	16	68	197

The six functional groups involve transcription factors, phytohormones, cell cycle regulators, defence/stress responders, kinases and ubiquitin/26 S proteasome factors in different expression patterns (shown in Figure 5). Raw data for the clusters are in Additional file 7.

#### Transcription factors

772 preferentially expressed transcription factor (TF)-related transcripts in 55 distinct TF families showed a significant skew to 15 families: MYB/MYB-related, AP2-EREBP, C2H2, bHLH, NAC, Orphan, MADS, HB, C3H, bZIP, ABIVP1, GRAS, SET, WRKY and GRAS (Table 1 and Additional file 7a). Overall, the number of TF transcripts showing late accumulation patterns (c0, c1 and c2) was greater than that showing middle (c3) and early (c4) accumulation patterns, which indicates that late development and maturation phases may need more TFs than the early phase. Transcripts of distinct families showed heterogeneous distribution in the five clusters. For example, most AP2-EREBP, bHLH, MYB/MYB-related and orphan transcripts were in c0, c1 and c2 clusters. C3H transcripts were mainly distributed in c1 and c4 clusters, SET and HMG transcripts were mainly in c4, and CSD and TAZ transcripts were only in c4. Multiple TF genes with distinct expression features were also identified in developing *Arabidopsis *pollen [13,14] and developing maize anthers [29]. And sperm cells of *Arabidopsis *pollen had their unique TFs [15]. The transcriptome of mature soybean showed an enrichment of TFs [13,14]. This finding suggested that different development events may require different TFs and/or a combination of TFs from distinct families.

#### Plant hormones

146 hormone-related transcripts showed pollen-preferential expression (Table 1 and Additional file 7b), and most (107) were associated with auxin (80) and ethylene (27) signalling, which indicates the importance of auxin and ethylene signalling in pollen development and germination/tube growth. The ethylene-related transcripts were mainly distributed in c0 and c1 clusters (19/27), whereas the auxin-related transcripts showed a relatively even distribution in all five clusters with larger numbers in c1 and c2. This difference in expression patterns suggests that ethylene may be implicated mainly in pollen maturity and germination, whereas auxin signalling may have more diverse functions in addition to possible roles in maturation and germination (see Discussion). As well, some transcripts related to gibberellin, brassinosteroid and jasmonic acid signalling showed distinct expression patterns (Table 1). Expression profile analyses of developing rice anthers by 10 K [30] and 44 K microarray analysis [31] implied the important role of multiple phytohormones, such as gibberellins, anuxin and ethylene, in pollen development.

#### Ubiquitin/26S proteasome system

Our analysis revealed 603 UPS transcripts involving distinct UPS components with pollen-preferential expression, and 406 of them encode F-box proteins (Additional file 7)c, which have a crucial role of conferring specificity on the UPS for appropriate targets. These F-box transcripts had distinct expression patterns. The highest number of the F-box transcripts (108) showed an early accumulation pattern (c4), with relatively high distribution in c1, whereas the remaining transcript were distributed in c0, c2 and c3 clusters. Other transcripts of UPS components showed a distribution in the 5 clusters similar to that of F-box transcripts (Table 1).

#### Kinases

666 pollen-preferentially expressed transcripts encoded a kinase domain-containing protein; examples are receptor kinases/receptor-like kinases (197), calcium signal-related kinases (33), phospholipid signal-related kinases (39), mitogen-activated protein kinase (MAPK) kinases (16), PTOs/PTIs (7) and wall-associated kinases (WAKs, 37) (Table 1 and Additional file 7d). Most of the kinase transcripts accumulated to the highest level in GPGs (c0, 110), MPGs and GPGs (c1, 319) or MPGs (c2, 116), with relatively few showing a high accumulation in BCP and TCP (c3, 78) or UNMs (c4, 43). Consistent with calcium signals playing important roles in germination and polar tube growth, calcium signal-related kinase transcripts were mainly distributed in c1. Similarly, most inositol phosphate kinases, PTOs/PTIs and receptor kinase transcripts were in c1.

#### Cell cycle-related transcripts

197 cell cycle-related transcripts were expressed preferentially in pollen (Table 1 and Additional file 7e). Of these, 68 were expressed at the highest levels in UNMs and thereafter sharply downregulated (c4, 43) or still showed the highest accumulation in BCP (c3, 25) (Table 1). These transcripts included cell cycle core regulators (cdc2, CYCK1 and CYCB), cell cycle entry/exit-regulated proteins (APC components), cullin, prohibitin, anti-silencing function 1 (ASF1), FtsZ, FtsJ, MinD, GlsA and Hsp70. Interestingly, 99 cell cycle-related transcripts showed the highest accumulation in GPGs (c0, 36) or MPGs and GPGs (c1, 63).

#### Defense/stress response-related transcripts

474 defense/stress response-related transcripts were preferentially expressed in pollen (Table 1 and Additional file 7f). Of these, 82% (388/474) accumulated from a low level in UNMs to the highest level in MPGs and/or GPGs (c0, c1 and c2) (Table 1). These late-accumulating transcripts were largely abiotic/biotic stress-response proteins such as disease-resistant NB-NRC, NBS-LRR and pathogenesis-related proteins and other stress-response proteins, such as cold acclimation protein COR413-PM1, harpin-induced protein and wound-induced protein WI12 (Additional file 7f). This finding indicates that a global ability to deal with abiotic and biotic stress formed during pollen maturity may be essential to successful fertilization.

### A comparison of rice and *Arabidopsis *pollen transcriptomes

**Figure 6:**
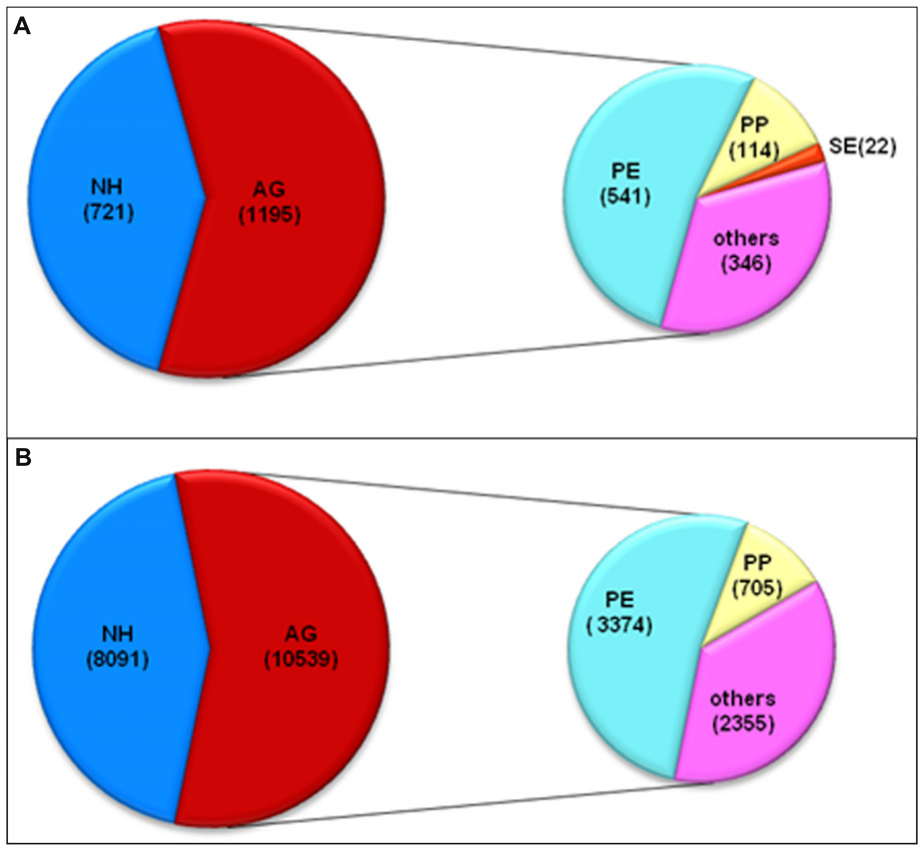
**Comparison of development stage-enriched (A) and pollen preferential genes (B) in rice and *Arabidopsis***. The large pie shows the numbers of rice development stage-enriched (A) or pollen-preferential genes (B) with or without homologs in the *Arabidopsis *genome. **NH**, no homologs; **AG**, having homologs. The small pie shows the numbers of the homologs in different expression patterns in *Arabidopsis*. **PE**, expression in pollen; **PP**, pollen-preferential expression; **SE**, pollen stage-enriched expression; **others**, undetectable expression in pollen. The transcriptome data of developing pollen of *Arabidopsis *were from [14]. Detailed information is in Additional file 8.

Based on published data of transcriptomes of mature or developing pollen of *Arabidopsis *[14], we compared possibly conserved and diverse features of rice and *Arabidopsis *pollen transcriptomes using pollen-preferential and development stage-enriched transcript datasets. By a BLAST searching with a cutoff of E-value < 1.0E-05, we revealed that 62.4% (1195 genes) of 1916 stage-enriched genes in rice pollen have conserved counterparts in the *Arabidopsis *genome; 677 (containing 22 stage-enriched genes) of these homologous genes were expressed in developing *Arabidopsis *pollen (Figure 6 and Additional file 8b). Consistent with this result, further analysis of rice pollen preferential genes showed that 56.6% of rice pollen-preferential genes (10,539 of 18,630) had homologous partners in the *Arabidopsis *genome, corresponding to 6434 genes in *Arabidopsis *genome (Figure 6 and Additional file 8a). However, among the 6434 genes, 63.4% (4079 containing 705 pollen-preferential genes) were expressed in developing pollen of *Arabidopsis *(Figure 6 and Additional file 8a). Furthermore, we used TFs, which are key regulators of different development events, as examples to compare conserved features of TFs. Of the 772 rice pollen-preferential TF transcripts (also see above, and Additional file 9), 189 from 32 TF families had homologous sequences expressed in *Arabidopsis *pollen (cutoff: e-value < 1.0E-05), which implies their conserved functions in monocots and dicots. 17 transcripts from 5 families, including ARR-B, GRF, SBP, Trihelix and ZIM, did not have corresponding homologous partners in the *Arabidopsis *dataset, although other members of the 5 families are expressed in developing *Arabidopsis *pollen (Table 2). Interestingly, 13 TF families, such as CSD, DBP and DDT, appeared to be specific to rice pollen, and none were identified in developing *Arabidopsis *pollen (Table 2); whereas 7 families, such as AtRKD, CAMTA and REM which expressed in *Arabidopsis *pollen, were not identified in the rice pollen transcriptome dataset (Table 2).

**Table 2:** Comparison between rice and *Arabidopsis *pollen-expressed transcription factors.

		Rice				*Arabidopsis*			
		
Family	NC	NWG	NGC	pollen		NWG	NGC	pollen	
							
				No.	%			No.	%
ABI3VP1	5	52	48	24	50	11	11	4	36
Alfin-like	2	9	9	5	56	7	7	6	86
AP2-EREBP	17	164	146	63	43	138	125	43	34
ARF	4	25	25	8	32	23	19	8	42
ARID	1	5	5	2	40	7	6	6	100
ARR-B		8	8	1	13	15	12	6	50
AtRKD		-	-	-		5	2	2	100
BBR/BPC		-	-	-		7	6	5	83
BES1/BZR	1	6	6	3	50	6	5	4	80
bHLH	8	144	133	51	38	164	112	42	38
bZIP	9	85	80	25	31	73	67	34	51
C2C2	6	81	78	27	35	103	83	31	37
C2H2	19	102	97	54	56	212	162	102	63
C3H	5	66	65	33	51	167	125	80	64
CAMTA		-	-	-		6	6	6	100
CCAAT	8	45	44	18	41	35	32	20	63
CPP	2	11	11	5	45	8	6	4	67
CSD		2	2	1	50	-	-	-	
DBP		6	6	4	67	-	-	-	
DDT		6	6	5	83	-	-	-	
G2-like	2	46	46	11	24	40	36	17	47
GeBP	2	6	5	2	40	16	7	6	86
GRAS	9	54	49	20	41	33	32	16	50
GRF		9	9	1	11	9	8	4	50
HB	11	91	83	32	39	91	84	31	37
HMG		9	9	4	44	-	-	-	
HRT	1	1	1	1	100	3	3	3	100
HSF	1	25	25	6	24	21	21	6	29
Jumonji	1	15	15	8	53	5	5	5	100
LFY		1	1	1	100	-	-	-	
LIM		6	6	3	50	-	-	-	
LUG		6	5	1	20	-	-	-	
MADS	19	64	59	44	75	109	79	30	38
MYB	13	121	118	51	43	131	123	43	35
MYB-like	14	77	69	32	46	67	56	37	66
NAC	4	123	116	51	44	96	88	32	36
Orphans	1	132	106	45	42	2	2	1	50
PHD	3	49	49	20	41	11	10	9	90
PLATZ		12	12	3	25	-	-	-	
Pseudo ARR-B			5	5	1	20	-	-	-
RAV		-	-	-		11	10	2	20
REM		-	-	-		21	14	3	21
RWP-RK/NLP	1	12	12	5	42	9	9	4	44
S1Falike		2	2	2	100	-	-	-	
SBP		19	17	4	24	16	15	5	33
SET	2	31	30	20	67	46	40	36	90
SNF2		36	33	18	55	-	-	-	
TAZ		5	5	1	20	-	-	-	
TCP	4	22	21	10	48	26	20	10	50
Trihelix		19	17	5	29	29	27	16	59
TUB	6	14	14	6	43	10	10	9	90
ULT		2	2	2	100	-	-	-	
VOZ		-	-	-		2	2	1	50
Whirly		-	-	-		3	3	3	100
WRKY	6	97	89	20	22	72	62	33	53
zf-HD	2	15	12	7	58	15	14	3	21
ZIM		16	16	6	38	2	1	1	100

**Total**	**189**	**1959**	**1827**	**772**	**42**	**1883**	**1567**	**769**	**49**

NC, number of transcription factors (TFs) conserved in rice and *Arabidopsis *pollen; NWG, number of diverse TFs transcripts in whole genome; NGC, number of diverse TFs transcripts in the Genechip. "-", indicating undetectable expression of the corresponding genes in *Arabidopsis *or rice pollen. Data for *Arabidopsis *pollen-expressed TFs gens were from Honys and Twell (2004). For details, see Additional file 9.

### Analysis of *cis*-acting regulatory elements from rice pollen stage-enriched genes

To provide insights into the transcription regulation of gene expression during rice pollen development, we identified *cis*-acting regulatory elements from 2-kb regions upstream of the start codon of the development stage-enriched genes using the PLACE database. Of 170 identified *cis*-elements, 107 (62.9%) had unknown functions (Additional file 10), which suggests that they represent the new *cis*-element in regulating transcription during rice pollen development. Among the known *cis*-elements, GTGANTG10 and POLLEN1LELAT52 are known pollen-specific *cis*-elements, identified in BCP- and TCP-enriched genes, respectively, and also identified in rice male gamete- and tapetum-specific genes as revealed by LM-microrray [32]. Impressively, a high proportion of respective UNM-, TCP- and MPGs-enriched genes shared common *cis*-elements, such as CAGATAA in UNMs, CACGTG in BCP, AAATAAG in TCP, and ATATAT in MPGs. However, relative to the "U" type distribution of stage-enriched genes, the diversity of identified *cis*-elements showed a nearly reversed distribution, and more *cis*-elements were found in BCP. Additionally, the most cis-elements were identified in GPGs. The *cis*-elements identified in respective stage-enriched genes appeared different. The results imply that different regulators or a combination of regulators are involved in regulation of pollen development at respective stages, and BCP and GPGs may require more diverse regulators than do other pollen.

## Discussion

Our analysis of the genome-wide gene expression profiles of developing and germinated rice pollen revealed dynamic characteristics of transcriptomes during pollen development and germination, and led to the identification of 25,062 transcripts expressed preferentially in rice pollen among the 51,279 transcripts on the array. Of these, 2,203 showed development stage-enriched expression. Furthermore, the pollen-preferential transcripts involved in 6 functional groups -- cell cycle regulators, phytohormones, kinases, UPS, transcription factors, and defense/stress responders -- could be classified into 5 expression patterns. The transcripts from distinct functional groups showed heterogeneous distribution in the 5 expression patterns, which suggests that the change in expression profiles were associated with the requirement of different events in pollen development and germination. These data provide information on a large number of candidate genes for further elucidation of the molecular mechanism underlying pollen development and germination and for use in control of pollen fertility and crop yield in rice.

### Conserved and divergent features of the transcriptome of developing pollen between rice and *Arabidopsis*

Rice and *Arabidopsis *are the best-characterized experimental models for eudicot and monocot plants, respectively, two major evolutionary lineages within the angiosperms. The developing pollen from microspore to mature stages in rice and *Arabidopsis *shares important cellular events, and their mature pollen grains are tricellular [33]. Consistent with the common cellular features, the diversity of genes expressed during pollen development from microspore to mature pollen stages was greatly decreased in both two species. The development stage-enriched genes showed a similar change tendency in developing rice and *Arabidopsis *pollen (for details, see below), and had a similar distribution in most functional terms (Additional file 11, detailed in Additional file 5a and b for rice and Additional file 12 for *Arabidopsis*). The rice pollen transcriptome had homologs of several genes such as *DUO3 *(At1g64570), *FBL17 *(At3g54650) and *GEX1 *(At2g35630) (Additional file 8) which are key regulators of germline development in *Arabidopsis *[1].

However, developing rice pollen appeared to express more development stage-enriched transcripts associated with defence/stress response in MPGs than do *Arabidopsis *pollen. Rice pollen at bicellular stage showed enrichment of more signalling-related transcripts than that in other stages as compared with *Arabidopsis *pollen, which showed signalling-related transcripts enriched in MPGs (Additional file 11). As well, these functional features of the transcription/RNA process seemed to greatly differ in developing pollen of rice and *Arabidopsis *(Additional file 11). This finding suggested a possible difference between pollen development in rice and *Arabidopsis *in the mechanism to handle defence/stress response, signalling and gene expression regulation. Consistent with this notion, our analysis showed 56.6% of rice pollen-preferential genes had homologs in the *Arabidopsis *genome, a finding similar to that from genome-wide comparison of rice and *Arabidopsis *or comparison of genes expressed in different organs [34]. However, besides the fact that 43.4% of rice pollen- preferential genes had no homologs in *Arabidopsis *genomes, a high proportion of genes conserved in *Arabidopsis *genome were not detected to express in *Arabidopsis *pollen (Additional file 8). These features, in combination with the finding that rice pollen preferentially expressed a set of unique TFs as compared with *Arabidopsis *pollen (Table 2), suggest a difference between rice and *Arabidopsis *in molecular regulation associated with pollen development.

### A shift in gene expression profiles during pollen development is associated with the requirement of distinct cellular events

We showed a greatly decreased diversity of transcripts in developing rice pollen from UNMs to MPGs, which is consistent with the observations in developing pollen in *Arabidopsis *[14]. In contrast to this finding, the number of development stage-enriched transcripts showed a "U-type" change during the development process, with the lowest in BCP. A similar "U-type" change tendency can be observed for stage-enriched transcripts of developing *Arabidopsis *pollen by re-analyzing *Arabidopsis *microarray data now available. These results suggest that a shift in gene expression program may exist in pollen development and BCP may be a key point for the regulation of the shift. This notion was also supported by the following evidence. First, several early observations in different plant species showed that developing pollen expressed distinct early and late transcript populations at early and late stages, respectively [18]. Second, protein profiles were found to be different in early and late pollen [35]. Third, the diversity of transcripts from distinct functional groups both stage-enriched and stage-downregulated displayed stage-dependent changes. For example, relatively more stage-enriched cell cycle-related genes were in UNMs than in other stages, and BCP had more signalling-related transcripts than other pollen, whereas UNMs had more stage-downregulated transcripts implicated in signalling, and cell cycle-related transcripts were greatly downregulated in BCP (Figure 3). As development advanced, transportation-related transcripts displayed statistical importance in TCP (Figure 4), and diverse wall-related and defence/stress-related transcripts accumulated to a high level during pollen maturity (Figure 3). Finally, relative to the smallest set of development stage-enriched genes at BCP, more diverse *cis*-elements were identified in the set as compared with the UNM and TCP sets (Additional file 10). Taken together, these data suggest that the shift may be essential to changes in development events from cell division and differentiation at early stages to maturation at late stages.

### Germinated pollen has a gene expression pattern similar to that of mature pollen

Transcription and translation inhibition experiments have shown that germination and early polar tube growth strictly depend on protein synthesis and are relatively independent of transcription in numerous plant species [14,18-20]. Transcriptome research of *Arabidopsis *pollen also suggested that the transcriptome of mature pollen skews toward pollen germination and tube growth [14]. Analysis of expression profiles of anther-expressed genes in rice indicated that genes possibly implicated in germination and pollen tube elongation are accumulated in late stages [32]. However, direct molecular evidence was lacking. Our data showed GPGs had a transcriptome profile most similar to MPGs (99%) (Additional file 3). Together with the proteome observations that the protein expression profiles of GPGs and MPGs mainly show variation in expression levels in rice [36] and *Gymnospermae pine *[37], our data clearly indicate that pollen germination and early tube growth mainly depend on these pre-synthesized mRNAs in mature pollen, at least in rice. In addition, our results showed several genes were up- or downregulated during germination (c0 and c2), and GPGs had a set of enriched transcripts. Therefore, these transcriptionally changed and GPG-enriched genes could be involved mainly in late tube growth and interaction with stigma cells and possibly play roles in germination and early tube growth. However, a recent transcriptome study demonstrated that hydrated pollen and pollen tubes have a larger number of newly expressed genes than do mature pollen [21]. This distinction may be associated with the different characteristics of rice and *Arabidopsis *pollen, but detailed studies are needed.

### Pollen development and germination are stringently regulated by 26S proteasomes

As an important molecular feature, rice pollen preferentially accumulated large numbers of UPS transcripts (Additional file 7c). Importantly, among these transcripts, 406 transcripts (369 unigenes) encode F-box proteins, which represent more than half of the now-predicted potential F-box proteins (687) in the rice genome [38]. F-box proteins are considered to function as a key component of E3 ligase complexes to specifically recognize proteins targeted for degradation by UPS. Two E3 complexes, APC and Skp1/Cullin/F-box (SCF), are known to control cell cycle progression [39] by regulating the periodically selective degradation of cyclins and other cell cycle regulators such as CDC6 [40], CDT1a [41], E2Fc [42], and the CDK inhibitor ICK2/KRP2[43-45]. Most of the cell-cycle regulator transcripts mentioned above accumulated at the highest level in UNMs and were co-expressed with several F-box transcripts involved in APC, skp1 and cullin (Table 1 and Additional file 7e). This finding suggests that early-accumulated F-box transcripts are implicated in cell cycle regulation. However, our data showed 108 F-box transcripts accumulated at the highest level in UNMs. This preferential presence of multiple F-box transcripts in UNMs may imply other functions of F-box proteins besides cell cycle control.

In addition, 249 F-box transcripts showed preferential accumulation in pollen following completion of the cell cycle (Table 1). Accordingly, many F-box transcripts are enriched in sperm cells of *Arabidopsis *[15]. Although functions of F-box genes in pollen maturity, germination and tube growth still remain unidentified, several studies have revealed that in self-incompatible plants such as *Antirrhinum hispanicum, *the pollen-expressed F-box protein AhALF-S2 acts as a pollen determinant to control pollen function in the self-incompatible reaction [46], and UPS also has roles in regulating polarized cell morphogenesis [47]. *In vitro *experiments showed that inhibition of UPS activity strongly inhibited pollen germination in kiwifruit [48]. Together, these data indicate that multiple F-box transcripts, which are preferentially accumulated in pollen, are involved in pollen maturity, germination and tube growth after the completion of the cell cycle.

### Cell cycle transcripts show distinct early and late accumulation patterns

The formation of tricellular pollen involves PMI and PMII, and the fate of daughter cells is determined after PMI [1,5]. Consistent with this cellular feature, transcripts encoding key cell cycle proteins, including cdc2, CYCK1, CYCB, ASF1, FtsZ, MinD, GlsA and Hsp70, were accumulated preferentially in UNMs (Additional file 7e). Generally, during the cell cycle, different CDK/cyclin complexes activate substrates functioning in the G1-to-S and G2-to-M transition and then trigger the onset of DNA replication and mitosis, respectively [49]. CDKs (cdc2) and cyclins are core regulators in the cell cycle, and the activity of CDK/cyclin complexes is controlled by several regulators [6,45,49]. The histone chaperone ASF1 is predicted to participate in DNA damage repair and histone acetylation in the mitotic S phase, and depletion of ASF1 results in the accumulation of S-phase cells [50-52]. FtsZ is required for formation of the division ring in bacterial cell division [53], and the position of the FtsZ-based division ring is mainly determined by MinD [54,55]. The ring is involved in the position determination of cell division. GlsA and Hsp70 interact as partner chaperones to regulate asymmetric division in *Volvox carteri *[56,57]. These results suggest that these proteins and/or their interactions would be implicated in regulation of pollen cell cycle. However, we found most of these key cell-cycle protein transcripts were downregulated as pollen entered the BCP stage. Most of the preferentially expressed cell cycle-related transcripts in BCP and/or TCP were those encoding general enzyme proteins such as serine/threonine protein phosphatases PP2 and PP1, dihydrolipoamide dehydrogenase, and nucleoside diphosphate kinase (Additional file 7e). This result implies that these key cell-cycle protein transcripts expressed preferentially at the UNM phase may be essential for PMII, possibly through protein dephosphorylation/phosphorylation.

Impressively, large numbers of transcripts encoding cell-cycle core regulators CYCA, CYCB, CYCC and CDC2, mitosis checkpoint proteins, and mitotic entry/exit-related proteins cullin, skp1, Mob1, TDP and RAD23 were accumulated preferentially in MPGs and/or GPGs (Additional file 7e). A similar expression pattern was observed in *Arabidopsis *and soybean mature pollen [11,13]. In *Arabidopsis*, the sperm cells are in S-phase at anthesis, continue the cell cycle process during pollen tube growth and reach G2 just before fertilization, whereas the vegetative nucleus is arrested in G1[1]. The above results suggest these pre-synthesized cell-cycle transcripts at pollen maturity have roles in mitotic progression after fertilization.

### Kinases and phytohormones function in pollen germination and polar tube growth

Most of the transcripts encoding receptor/receptor-like kinases and those encoding calcium and phospholipid signalling-related kinases, MAPKs, WAKs and PTOs/PTIs displayed late accumulation patterns, with the highest levels in MPGs and/or GPGs (Table 1). Specifically, 87% of the identified pollen-preferential receptor kinase transcripts accumulated to the highest levels in MPGs and/or GPGs (Table 1). Calcium signal and calcium concentration gradients play important roles in pollen germination and polar tube growth [58,59]. Phosphoinositide kinases participate in the regulation of cytosolic Ca^2+ ^concentration by promoting Ca^2+ ^sequestration or mobilizing intracellular Ca^2+ ^stores [60]. Re-organization of the cytoskeleton is one of the early events after hydration of mature pollen grains, and MAP affects the dynamics of microtubule cytoskeleton by phosphorylating microtubule-associated proteins [61]. Receptor kinases are required for pollen maturity, tube growth and pollen-stigma interaction [62-64]. Together, these data suggest that late accumulated kinase transcripts are essential for pollen germination, tube growth and pollen-stigma interaction.

The importance of auxin and ethylene in pollen germination and tube growth has been a focus for a long time. In *Arabidopsis*, free IAA is lacking in very young pollen but is accumulated to extremely high levels in mature and germinated pollen; high IAA level is involved in the control of pollen tubes growth towards the egg cell in the ovule [65]. In rice, IAA is accumulated in anthers containing tricellular pollen [31]. Study of *Torenia fournier *revealed the roles of IAA in the increase of secretory vesicles, in enhanced synthesis of pectin and in the decrease of cellulose density in pollen tubes [66]. In rice pollen, 75% of the pollen-preferential auxin synthesis/signalling-related transcripts, which involve auxin-conjugated hydrolysis, efflux-carrier, transport and synthesis proteins (enzymes) (Additional file 7b), showed late accumulation patterns (c0, c1 and c2). The expression profile seems compatible with the high level accumulation of free IAA observed in *Arabidopsis *pollen [65] and mature rice anthers [31]. These lines of evidence suggest that changes in free IAA levels involve the combined effects of auxin biosynthesis, conjugation and transport during pollen development and maturity.

Ethylene is also required for pollen germination and tube growth because inhibitors of ethylene biosynthesis strongly retard pollen germination and tube growth [67-69]. Although several observations reveal that auxin initiates ethylene production in pollen [67], current data show that pollination-mediated initial burst of ethylene in the pistil regulates early tube growth [68,69]. Our study revealed 81% (22/27) of pollen-preferential ethylene signalling-related transcripts accumulated at the highest level in MPGs and/or GPGs, and most of them encode the components of ethylene signalling rather than ethylene synthesis enzymes such as ACC synthase and ACC oxidase (Additional file 7b). These data appear to be compatible with the model of auxin action in pollination, which assumes that auxin from pollen diffuses into the pistil, where auxin stimulates the production of ethylene, which in turn triggers pollen germination/tube growth and ovary development. This finding suggests that ethylene signalling may be required for pollen tube growth and coordination between pollen tubes and pistil cells by interacting with auxin signalling. This notion seems to be supported by the concordant expression of auxin and ethylene signalling transcripts in MPGs and GPGs in rice and the observation that ovary development and pollen germination/tube growth are coordinately regulated by auxin and ethylene following pollination in orchid [67].

## Conclusions

We analyzed the dynamic changes in genome-wide gene expression profiles of rice pollen during 5 sequential development stages, from microspore to germination. Overall, pollen development from microspores to mature pollen is associated with a great decrease in diversity of transcripts and the "U-type" change in the number of stage-enriched transcripts, with the lowest at the bicellular pollen stage. These features were conserved in the transcriptome of developing pollen of *Arabidopsis*. The gene expression profile of germinated pollen was similar to that of mature pollen. Our analysis also reveals that changes in functional groups of the stage-enriched and -downregulated transcripts and in expression patterns of pollen-preferential transcripts involving important regulatory proteins are compatible with the transition of distinct cellular events during pollen development and germination. A comparison showed that stage-enriched transcripts both in rice and *Arabidopsis *had similar distribution in most of functional terms but great difference in defence/stress response, signalling and transcription/RNA process. A proportion of rice pollen-preferential genes had no homologs in the *Arabidopsis *genome or their homologs were not expressed in *Arabidopsis *pollen. Several transcription factors were identified to be diverged in the two species. These data supply the first comprehensive and comparative molecular information for further understanding the mechanism underlying pollen development and germination.

## Methods

### Plant materials

Rice cultivar Zhonghua 10 (*Oryza sativa *L. ssp. *japonica*) was used for this study. Roots and leaves were collected from 2-week-old seedlings grown in a climate chamber under a 12-hr light/12-hr dark cycle at 28°C. Callus cells were induced from rice seeds on N6 solid medium containing 2,4-D (2 mg/L) in the dark at 25°C for approximately 1 month. Other materials described below were harvested from rice plants grown under natural conditions in the growing season (from May to September) in Beijing, China.

### Pollen isolation and purification

For pollen isolation, rice anther samples were first classified into uninucleate, bicellular, immature tricellular stages by the distance between auricles of the last two leaves and the length of the panicle and the floret [70,71]. Then, uninucleate mcirospores (UNMs), bicellular pollen (BCP), and immature tricellular pollen (TCP) were isolated from the classified anthers at the corresponding stages.

UNMs and TCP were prepared as follows: the anthers at the uninucleate and tricellular stages were crashed gently in 0.4 M mannitol at 4°C; the resulting slurry was filtered through 150 μm and subsequent 100 μm nylon mesh to remove anther debris, through 60 μm nylon mesh to remove cell debris, and finally through 30 μm nylon mesh to collect pollen. Bicellular pollen (BCP) was collected as described [14] with several modifications. Briefly, the anthers containing BCP were collected and gently ground in 0.4 M mannitol at 4°C. After filtering subsequently through 150 μm and 60 μm nylon mesh, the pollen was harvested by centrifugation at 500 *g *for 5 min at 4°C, and then purified by 25%/30%/45%/80% Percoll (Pharmacia) step gradient under 500 *g *centrifugation for 5 min at 4°C. Resulting BCP cells were collected from the 30%/45% fraction, pelleted by centrifugation at 500 *g *for 5 min at 4°C, and washed with 0.4 M mannitol. Mature pollen grains (MPGs) were collected at anthesis stage [22]. Germinated pollen grains (GPGs) were obtained as described [22,36]. The purity of the isolated pollen was determined by examination on light microscopy (Carl Zeiss) after 4',6-diaminophenylindole (DAPI) (Molecular Probes) staining, and viability of mature pollen was assessed by fluorescein 3',6'-diacetate (FDA) staining [72]. Germination ratio of mature pollen grains was evaluated on microscopy after culture in germination medium *in vitro *(Carl Zeiss).

### RNA extraction

Total RNA was extracted from sporophytic tissues and isolated pollen at individual development stage by use of RNAplant reagents (Tiangen Biotech) and purified by use of the RNeasy Plant Kit (Qiagen) according to the manufacturer's instruction. The yield and purity of RNA were determined spectrophotometrically (Beckman Coulter DU640).

### Affymetrix GeneChip hybridization and data analysis

For Affymetrix GeneChip analysis, 8 μg of total RNA was used for making biotin-labeled cRNA targets. All the procedures for cDNA and cRNA synthesis, cRNA fragmentation, hybridization, washing and staining, and scanning were conducted according to the GeneChip Standard Protocol (Eukaryotic Target Preparation, Affymetrix). The poly-A RNA Control and One-Cycle cDNA Synthesis kits were used in this experiment as described at the website http://www.affymetrix.com/products/arrays/specific/rice.affx. Information about the GeneChip^® ^Rice Genome Array (MAS 5.0) could be accessed from the Affymetrix website http://www.affymetrix.com/products/arrays/specific/rice.affx. GCOS software (Affymetrix GeneChip Operating Software) was used for data collection and normalization. Overall intensities of all probe sets of each array were scaled to 500 to guarantee that hybridization intensity of all arrays was equivalent, and each probe set was assigned a "P" (present), "A" (absent) or "M" (missing) value and *p*-value from algorithms in GCOS. Correlation coefficient values were calculated for replicate experiments of different tissues, and the correlation of means for different tissues from replicated experiments was calculated. The percentage of probe sets that were present ("P") in each array was listed and compared. To identify genes expressed preferentially in rice pollen, the *Z-*score transformation normalization method was used to compare expression levels of genes from pollen at individual stages and sporophytic tissues and to directly calculate significant changes in gene expression between different samples. Z-scores were calculated by dividing the difference between the pollen (X_i_, median of triplicates) and the sporophytic tissue (μ, mean) with the standard deviation (SD) of all of the other tissues by the following equation:

During Z-score calculation, we made a slight change by not including the value of specific stage of pollen for measuring μ and SD.

To identify probe sets expressed preferentially in pollen as compared with sporophytic tissues, we measured the relative ratio between pollen and other samples using the following equation:

where P, S, R and L represent the expression level of a given gene in pollen, callus cells, roots and leaves, respectively.

For identification of probe sets expressed preferentially in each stage of pollen development, all three distinct sporophytic tissues were used as controls, and the relative ratio was measured by use of the following equation:

where *Px, S, R, L *and *P *represent expression levels of genes in pollen at each stage, in callus cells, roots, leaves and a given pollen sample, respectively.

### Bioinformatics analyses

The molecular function and cellular component term "enrichment status" and "hierarchy" of pollen-enriched genes were analyzed by use of EasyGo software (a web server, http://bioinformatics.cau.edu.cn/easygo/) [73].

Expression pattern analysis was performed with the mean values of replicates with use of GeneCluster 2.0 http://www.broad.mit.edu/cancer/software/genecluster2/gc2.html, which allows for visualizing the profile of each cluster. After being normalized to mean 0 and variance 1, clusters were created with default parameters, except for cluster range 3-7. Different cluster ranges were compared, and the range of 5 was selected because the distribution of functional categories between clusters possessed the most significant difference (χ2 20 DF, 56.00, p ≤ 0.01).

### Real-time quantitative RT-PCR

Total RNA was prepared as described above from each of three independent biological samples for each material. Real-time quantitative RT-PCR was performed as described [27]. Briefly, 2.5 μg of total RNA was used for first-strand cDNA synthesis with use of ReverTra Ace (TOYOBO). The cDNA samples were diluted to 2.5 ng/μl. Triplicate quantitative assays were performed with use of the Stratagene Mx3000P system (Applied Biosystems) with 4 μL of each cDNA dilution and the Power SYBR Green Master mix (Applied Biosystems) according to the manufacturer's protocol. Gene-specific primers were designed by use of PRIMEREXRESS software (Applied Biosystems). The relative quantification method (delta-delta threshold cycle) was used to evaluate quantitative variation between the three independent replicates. Amplification of 18S rRNA was used as an internal control to normalize all data.

### *Cis*-elements analysis

The Element software http://element.cgrb.oregonstate.edu/element_about.html was used for *cis*-element search in the promoter regions of stage-enriched genes. In the database, the promoter sequences for rice genes were mainly from IRGSP Rice (*Oryza sativa *ssp. japonica). We selected a 2,000-bp promoter region in each gene to search for the possible *cis*-element using the TIGR locus ID. The background model statistics were derived from the frequencies of all possible 3-8 mer words in the upstream sequences of 34,967 non-transposable-element-related japonica rice genes (based on TIGR version 5) in Affymetrix rice microarrays.

## Authors' contributions

TW and YX designed the experiments and wrote the paper. LQW performed most of the experiments (pollen isolation and purification, RNA extraction, qRT-PCR, data analysis) and wrote the draft of the paper. WYX was responsible for the Affymetrix GeneChip hybridization and most of the data analysis. ZS analysed the data and designed the primers for qRT-PCR. ZYD revised and proofread the paper. All the authors read and approved the final manuscript.

## Supplementary Material

Additional file 1 Expression profiles of the 54 genes examined by quantitative RT-PCR. Click here for file

Additional file 2 The number of transcripts/genes expressed in rice and *Arabidopsis *pollen. Click here for file

Additional file 3 Correlation coefficients among transcriptome profiles from callus cells and pollen at each stage. Click here for file

Additional file 4 Transcripts with different expression patterns. Click here for file

Additional file 5 Stage-enriched and down-regulated transcripts and their functional categories in rice pollen. Click here for file

Additional file 6 Gene Ontology (GO) term "enrichment status" for the pollen stage down-regulated genes in MPGs and GPGs. Click here for file

Additional file 7 **Pollen-preferential transcripts of transcription factors (Additional file**7**a), phytohormones (Additional file**7**b**), **the unbiquitin/26 S proteasome system (Additional file**7c**), kinases (Additional file**7d**), cell cycle regulation (Additional file**7**e) anddefense/stress responders (Additional file**7**f) and their expression patterns.** Click here for file

Additional file 8 Comparison of pollen preferential and stage-enriched genes in rice with those in *Arabidopsis*. Click here for file

Additional file 9 Comparison of transcription factors expressed preferentially in rice and *Arabidopsis *pollen. Click here for file

Additional file 10 ***Cis***-**elements identified in the promoter regions of stage-enriched genes.** Click here for file

Additional file 11 Comparison of functional groups of stage-enriched genes during pollen development between rice and A*rabidopsis*. Click here for file

Additional file 12 Stage-enriched genes and their functional categories in *Arabidopsis *pollen. Click here for file

## References

[B1] Borg M Brownfield L Twell D Male gametophyte development: a molecular perspective Journal of experimental botany 2009 60 5 1465 1478 10.1093/jxb/ern355 19213812

[B2] McCormick S Control of male gametophyte development Plant Cell 2004 16 Suppl S142 153 10.1105/tpc.016659 15037731 PMC2643393

[B3] Ma H Molecular genetic analyses of microsporogenesis and microgametogenesis in flowering plants Annu Rev Plant Biol 2005 56 393 434 10.1146/annurev.arplant.55.031903.141717 15862102

[B4] Singh MB Bhalla PL Control of male germ-cell development in flowering plants Bioessays 2007 29 11 1124 1132 10.1002/bies.20660 17935220

[B5] Singh M Bhalla P Russell S Molecular repertoire of flowering plant male germ cells Sexual Plant Reproduction 2008 21 1 27 36 10.1007/s00497-008-0067-y

[B6] Brownfield L Hafidh S Durbarry A Khatab H Sidorova A Doerner P Twell D Arabidopsis DUO POLLEN3 is a key regulator of male germline development and embryogenesis Plant Cell 2009 21 7 1940 1956 10.1105/tpc.109.066373 19638475 PMC2729611

[B7] Brownfield L Hafidh S Borg M Sidorova A Mori T Twell D A plant germline-specific integrator of sperm specification and cell cycle progression PLoS genetics 2009 5 3 e1000430 10.1371/journal.pgen.1000430 19300502 PMC2653642

[B8] Twell D Oh SA Honys D Pollen Development, a Genetic and Transcriptomic View The Pollen Tube 2006 15 45 full_text

[B9] Becker JD Boavida LC Carneiro J Haury M Feijo JA Transcriptional profiling of Arabidopsis tissues reveals the unique characteristics of the pollen transcriptome Plant Physiol 2003 133 2 713 725 10.1104/pp.103.028241 14500793 PMC219046

[B10] Honys D Twell D Comparative analysis of the Arabidopsis pollen transcriptome Plant Physiol 2003 132 2 640 652 10.1104/pp.103.020925 12805594 PMC167004

[B11] Pina C Pinto F Feijo' JA Becker D Jr Gene family analysis of the Arabidopsis pollen transcriptome reveals biological implications for cell growth, division control, and gene expression regulation Plant Physiol 2005 138 744 756 10.1104/pp.104.057935 15908605 PMC1150393

[B12] Lee JY Lee DH Use of serial analysis of gene expression technology to reveal changes in gene expression in Arabidopsis pollen undergoing cold stress Plant Physiol 2003 132 517 529 10.1104/pp.103.020511 12805584 PMC166994

[B13] Haerizadeh F Wong CE Bhalla PL Gresshoff PM Singh MB Genomic expression profiling of mature soybean (Glycine max) pollen BMC Plant Biol 2009 9 25 10.1186/1471-2229-9-25 19265555 PMC2660330

[B14] Honys D Twell D Transcriptome analysis of haploid male gametophyte development in *Arabidopsis* Genome Biol 2004 5 11 R85 10.1186/gb-2004-5-11-r85 15535861 PMC545776

[B15] Borges F Gomes G Gardner R Moreno N McCormick S Feijo JA Becker JD Comparative transcriptomics of Arabidopsis sperm cells Plant Physiol 2008 148 2 1168 1181 10.1104/pp.108.125229 18667720 PMC2556834

[B16] Verelst W Twell D de Folter S Immink R Saedler H Munster T MADS-complexes regulate transcriptome dynamics during pollen maturation Genome Biol 2007 8 11 R249 10.1186/gb-2007-8-11-r249 18034896 PMC2258202

[B17] Cheung AY Wu HM Structural and signaling networks for the polar cell growth machinery in pollen tubes Annu Rev Plant Biol 2008 59 547 572 10.1146/annurev.arplant.59.032607.092921 18444907

[B18] Mascarenhas JP Molecular mechanisms of pollen tube growth and differentiation Plant Cell 1993 5 10 1303 1314 10.1105/tpc.5.10.1303 12271030 PMC160363

[B19] Hao H Li Y Hu Y Lin J Inhibition of RNA and protein synthesis in pollen tube development of *Pinus bungeana *by actinomycin D and cycloheximide New Phytol 2005 165 3 721 729 10.1111/j.1469-8137.2004.01290.x 15720683

[B20] Wang ML Hsu CM Chang LC Wang CS Su TH Huang YJ Jiang L Jauh GY Gene expression profiles of cold-stored and fresh pollen to investigate pollen germination and growth Plant Cell Physiol 2004 45 10 1519 1528 10.1093/pcp/pch174 15564535

[B21] Wang Y Zhang WZ Song LF Zou JJ Su Z Wu WH Transcriptome Analyses Show Changes in Gene Expression to Accompany Pollen Germination and Tube Growth in Arabidopsis Plant Physiol 2008 148 3 1201 1211 10.1104/pp.108.126375 18775970 PMC2577266

[B22] Dai S Li L Chen T Chong K Xue Y Wang T Proteomic analyses of *Oryza sativa *mature pollen reveal novel proteins associated with pollen germination and tube growth Proteomics 2006 6 8 2504 2529 10.1002/pmic.200401351 16548068

[B23] Fu JH Lei LG Chen LB Qiu GZ Wall ultrastructure and cytochemistry and the longevity of pollen of three grass species Australian Journal of Botany 2001 49 6 771 776 10.1071/BT00085

[B24] Song ZP Lu BR Wang B Chen JK Fitness estimation through performance comparison of F1 hybrids with their parental species *Oryza rufipogon *and *O. sativa* Ann Bot (Lond) 2004 93 3 311 316 10.1093/aob/mch036 PMC4242197 14724120

[B25] Zik M Irish VF Flower development: initiation, differentiation, and diversification Annual review of cell and developmental biology 2003 19 119 140 10.1146/annurev.cellbio.19.111301.134635 14570566

[B26] Russell SD Bhalla PL Singh MB Transcriptome-based examination of putative pollen allergens of rice (Oryza sativa ssp. japonica) Mol Plant 2008 1 5 751 759 10.1093/mp/ssn036 19825578

[B27] Li M Xu W Yang W Kong Z Xue Y Genome-wide gene expression profiling reveals conserved and novel molecular functions of the stigma in rice Plant Physiol 2007 144 4 1797 1812 10.1104/pp.107.101600 17556504 PMC1949881

[B28] Fu Y Wu G Yang Z Rop GTPase-dependent dynamics of tip-localized F-actin controls tip growth in pollen tubes J Cell Biol 2001 152 5 1019 1032 10.1083/jcb.152.5.1019 11238457 PMC2198818

[B29] Ma J Skibbe DS Fernandes J Walbot V Male reproductive development: gene expression profiling of maize anther and pollen ontogeny Genome Biol 2008 9 12 R181 10.1186/gb-2008-9-12-r181 19099579 PMC2646285

[B30] Wang Z Liang Y Li C Xu Y Lan L Zhao D Chen C Xu Z Xue Y Chong K Microarray analysis of gene expression involved in anther development in rice (Oryza sativa L.) Plant Mol Biol 2005 58 721 737 10.1007/s11103-005-8267-4 16158245

[B31] Hirano K Aya K Hobo T Sakakibara H Kojima M Shim RA Hasegawa Y Ueguchi-Tanaka M Matsuoka M Comprehensive transcriptome analysis of phytohormone biosynthesis and signaling genes in microspore/pollen and tapetum of rice Plant & cell physiology 2008 49 10 1429 1450 10.1093/pcp/pcn123 PMC2566925 18718932

[B32] Hobo T Suwabe K Aya K Suzuki G Yano K Ishimizu T Fujita M Kikuchi S Hamada K Miyano M Fujioka T Kaneko F Kazama T Mizuta Y Takahashi H Shiono K Nakazono M Tsutsumi N Nagamura Y Kurata N Watanabe M Matsuoka M Various Spatiotemporal Expression Profiles of Anther-Expressed Genes in Rice Plant Cell Physiol 2008 49 10 1417 1428 10.1093/pcp/pcn128 18776202 PMC2566926

[B33] Wilson ZA Zhang DB From Arabidopsis to rice: pathways in pollen development Journal of experimental botany 2009 60 5 1479 1492 10.1093/jxb/erp095 19321648

[B34] Ma L Chen C Liu X Jiao Y Su N Li L Wang X Cao M Sun N Zhang X Bao J Li J Pedersen S Bolund L Zhao H Yuan L Wong GK-S Wang J Deng XW Wang J A microarray analysis of the rice transcriptome and its comparison to Arabidopsis Genome Research 2005 15 1274 1283 10.1101/gr.3657405 16140994 PMC1199542

[B35] Bedinger PA Edgerton MD Developmental staging of maize microspores reveals a transition in developing microspore proteins Plant Physiol 1990 92 2 474 479 10.1104/pp.92.2.474 16667300 PMC1062316

[B36] Dai S Chen T Chong K Xue Y Liu S Wang T Proteomics identification of differentially expressed proteins associated with pollen germination and tube growth reveals characteristics of germinated *Oryza sativa *pollen Mol Cell Proteomics 2007 6 2 207 230 17132620 10.1074/mcp.M600146-MCP200

[B37] Fernando DD Characterization of pollen tube development in *Pinus strobus *(Eastern white pine) through proteomic analysis of differentially expressed proteins Proteomics 2005 5 18 4917 4926 10.1002/pmic.200500009 16247732

[B38] Jain M Nijhawan A Arora R Agarwal P Ray S Sharma P Kapoor S Tyagi AK Khurana JP F-Box proteins in rice. Genome-wide analysis, classification, temporal and spatial gene expression during panicle and seed development, and regulation by light and abiotic stress Plant Physiol 2007 143 4 1467 1483 10.1104/pp.106.091900 17293439 PMC1851844

[B39] Vodermaier HC APC/C and SCF: controlling each other and the cell cycle Curr Biol 2004 14 18 R787 796 10.1016/j.cub.2004.09.020 15380093

[B40] Castellano MM del Pozo JC Ramirez-Parra E Brown S Gutierrez C Expression and stability of Arabidopsis CDC6 are associated with endoreplication Plant Cell 2001 13 12 2671 2686 10.1105/tpc.13.12.2671 11752380 PMC139481

[B41] Castellano Mdel M Boniotti MB Caro E Schnittger A Gutierrez C DNA replication licensing affects cell proliferation or endoreplication in a cell type-specific manner Plant Cell 2004 16 9 2380 2393 10.1105/tpc.104.022400 15316110 PMC520940

[B42] del Pozo JC Boniotti MB Gutierrez C Arabidopsis E2Fc functions in cell division and is degraded by the ubiquitin-SCF^AtSKP2 ^pathway in response to light Plant Cell 2002 14 12 3057 3071 10.1105/tpc.006791 12468727 PMC151202

[B43] Lai J Chen H Teng K Zhao Q Zhang Z Li Y Liang L Xia R Wu Y Guo H Xie Q RKP, a RING finger E3 ligase induced by BSCTV C4 protein, affects geminivirus infection by regulation of the plant cell cycle Plant J 2009 57 5 905 917 10.1111/j.1365-313X.2008.03737.x 19000158

[B44] Liu J Zhang Y Qin G Tsuge T Sakaguchi N Luo G Sun K Shi D Aki S Zheng N Aoyama T Oka A Yang W Umeda M Xie Q Gu H Qu LJ Targeted degradation of the cyclin-dependent kinase inhibitor ICK4/KRP6 by RING-type E3 ligases is essential for mitotic cell cycle progression during *Arabidopsis *gametogenesis Plant Cell 2008 20 6 1538 1554 10.1105/tpc.108.059741 18552199 PMC2483368

[B45] Kim HJ Oh SA Brownfield L Hong SH Ryu H Hwang I Twell D Nam HG Control of plant germline proliferation by SCF(FBL17) degradation of cell cycle inhibitors Nature 2008 455 7216 1134 1137 10.1038/nature07289 18948957

[B46] Qiao H Wang H Zhao L Zhou J Huang J Zhang Y Xue Y The F-box protein AhSLF-S_2 _physically interacts with S-RNases that may be inhibited by the ubiquitin/26S proteasome pathway of protein degradation during compatible pollination in Antirrhinum Plant Cell 2004 16 3 582 595 10.1105/tpc.017673 14973168 PMC385274

[B47] Dittmar GA Wilkinson CR Jedrzejewski PT Finley D Role of a ubiquitin-like modification in polarized morphogenesis Science 2002 295 5564 2442 2446 10.1126/science.1069989 11923536

[B48] Speranza A Scoccianti V Crinelli R Calzoni GL Magnani M Inhibition of proteasome activity strongly affects kiwifruit pollen germination. Involvement of the ubiquitin/proteasome pathway as a major regulator Plant Physiol 2001 126 3 1150 1161 10.1104/pp.126.3.1150 11457965 PMC116471

[B49] Francis D The plant cell cycle--15 years on New Phytol 2007 174 2 261 278 10.1111/j.1469-8137.2007.02038.x 17388890

[B50] Sanematsu F Takami Y Barman HK Fukagawa T Ono T Shibahara K Nakayama T Asf1 is required for viability and chromatin assembly during DNA replication in vertebrate cells J Biol Chem 2006 281 19 13817 13827 10.1074/jbc.M511590200 16537536

[B51] Recht J Tsubota T Tanny JC Diaz RL Berger JM Zhang X Garcia BA Shabanowitz J Burlingame AL Hunt DF Kaufman PD Allis CD Histone chaperone Asf1 is required for histone H3 lysine 56 acetylation, a modification associated with S phase in mitosis and meiosis Proc Natl Acad Sci USA 2006 103 18 6988 6993 10.1073/pnas.0601676103 16627621 PMC1459006

[B52] Kats ES Enserink JM Martinez S Kolodner RD The Saccharomyces cerevisiae Rad6 postreplication repair and Siz1/Srs2 homologous recombination-inhibiting pathways process DNA damage that arises in asf1 mutants Mol Cell Biol 2009 29 19 5226 5237 10.1128/MCB.00894-09 19635810 PMC2747975

[B53] Lan G Daniels BR Dobrowsky TM Wirtz D Sun SX Condensation of FtsZ filaments can drive bacterial cell division Proc Natl Acad Sci USA 2009 106 1 121 126 10.1073/pnas.0807963106 19116281 PMC2629247

[B54] Zhang M Hu Y Jia J Gao H He Y A plant MinD homologue rescues Escherichia coli HL1 mutant (DeltaMinDE) in the absence of MinE BMC Microbiol 2009 9 101 10.1186/1471-2180-9-101 19457228 PMC2691406

[B55] Shen B Lutkenhaus J The conserved C-terminal tail of FtsZ is required for the septal localization and division inhibitory activity of MinC(C)/MinD Mol Microbiol 2009 72 2 410 424 10.1111/j.1365-2958.2009.06651.x 19415799 PMC2759774

[B56] Cheng Q Pappas V Hallmann A Miller SM Hsp70A and GlsA interact as partner chaperones to regulate asymmetric division in *Volvox* Dev Biol 2005 286 2 537 548 10.1016/j.ydbio.2005.08.028 16168403

[B57] Pappas V Miller SM Functional analysis of the Volvox carteri asymmetric division protein GlsA Mech Dev 2009 126 10 842 851 10.1016/j.mod.2009.07.007 19646527

[B58] Malho R Liu Q Monteiro D Rato C Camacho L Dinis A Signalling pathways in pollen germination and tube growth Protoplasma 2006 228 1-3 21 30 10.1007/s00709-006-0162-6 16937051

[B59] Cardenas L Lovy-Wheeler A Kunkel JG Hepler PK Pollen tube growth oscillations and intracellular calcium levels are reversibly modulated by actin polymerization Plant Physiol 2008 146 4 1611 1621 10.1104/pp.107.113035 18263780 PMC2287337

[B60] Xu J Brearley CA Lin WH Wang Y Ye R Mueller-Roeber B Xu ZH Xue HW A Role of Arabidopsis Inositol Polyphosphate Kinase, AtIPK2a, in Pollen Germination and Root Growth Plant Physiol 2005 137 1 94 103 10.1104/pp.104.045427 15618435 PMC548841

[B61] Heberle-Bors E Voronin V Touraev A Sanchez Testillano P Risueño MC Wilson C MAP kinase signaling during pollen development Sex Plant Reprod 2001 14 1 15 19 10.1007/s004970100087

[B62] Kaothien P Ok SH Shuai B Wengier D Cotter R Kelley D Kiriakopolos S Muschietti J McCormick S Kinase partner protein interacts with the LePRK1 and LePRK2 receptor kinases and plays a role in polarized pollen tube growth Plant J 2005 42 492 503 10.1111/j.1365-313X.2005.02388.x 15860008

[B63] Becraft PW Receptor kinase signaling in plant development Annu Rev Cell Dev Biol 2002 18 163 192 10.1146/annurev.cellbio.18.012502.083431 12142267

[B64] Shiu SH Bleecker AB Plant receptor-like kinase gene family: diversity, function, and signaling Sci STKE 2001 2001 113 RE22 10.1126/stke.2001.113.re22 11752632

[B65] Aloni R Aloni E Langhans M Ullrich CI Role of auxin in regulating *Arabidopsis *flower development Planta 2006 223 315 328 10.1007/s00425-005-0088-9 16208486

[B66] Wu JZ Lin Y Zhang XL Pang DW Zhao J IAA stimulates pollen tube growth and mediates the modification of its wall composition and structure in *Torenia fournieri* J Exp Bot 2008 59 9 2529 2543 10.1093/jxb/ern119 18544613 PMC2423660

[B67] Zhang XS O'Neill SD Ovary and gametophyte development are coordinately regulated by auxin and ethylene following pollination Plant Cell 1993 5 4 403 418 10.1105/tpc.5.4.403 12271070 PMC160280

[B68] Martinis DD Cotti G Hekker StL Harren FJM Mariani C Ethylene response to pollen tube growth in *Nicotiana tabacum *flowers Planta 2002 214 806 812 10.1007/s00425-001-0684-2 11882951

[B69] Holden MJ Marty JA Singh-Cundy A Pollination-induced ethylene promotes the early phase of pollen tube growth in *Petunia inflata* J Plant Physiol 2003 160 3 261 269 10.1078/0176-1617-00929 12749083

[B70] Itoh J Nonomura K Ikeda K Yamaki S Inukai Y Yamagishi H Kitano H Nagato Y Rice plant development: from zygote to spikelet Plant Cell Physiol 2005 46 1 23 47 10.1093/pcp/pci501 15659435

[B71] Kerim T Imin N Weinman JJ Rolfe BG Proteome analysis of male gametophyte development in rice anthers Proteomics 2003 3 5 738 751 10.1002/pmic.200300424 12748952

[B72] Eady C Lindsey K Twell D The significance of microspore division and division symmetry for vegetative cell-specific transcription and generative cell differentiation Plant Cell 1995 7 1 65 74 10.1105/tpc.7.1.65 12242352 PMC160765

[B73] Zhou X Su Z EasyGO: Gene ontology-based annotation and functional enrichment analysis tool for agronomical species BMC genomics 2007 8 246 10.1186/1471-2164-8-246 17645808 PMC1940007

