# Supplementary material for: Genome-scale analysis and comparison of gene expression profiles in developing and germinated pollen in Oryza sativa
Source: BMC Genomics. 2010 May 28;11:338. doi: 10.1186/1471-2164-11-338 (PMC2895629; doi:10.1186/1471-2164-11-338)
Supplement: Additional file 3 — Correlation coefficients among transcriptome profiles from callus cells and pollen at each stage. [file 1471-2164-11-338-S3.DOC]

**Additional file 3: Correlation coefficients among transcriptome profiles from callus cells and pollen at each stage.**

|  | **UNM** | **BCP** | **TCP** | **MPG** | **GPG** |
| --- | --- | --- | --- | --- | --- |
| **Callus cells** | 0.72 | 0.59 | 0.29 | 0.11 | 0.12 |
| **UNM** |  | 0.82 | 0.40 | 0.13 | 0.13 |
| **BCP** |  |  | 0.69 | 0.31 | 0.30 |
| **TCP** |  |  |  | 0.76 | 0.75 |
| **MPG** |  |  |  |  | **0.99** |

The average values of three independently replicated experiments for each sample were used for correlation analysis. The numbers represented the correlation coefficients (r-value) between a pairwise pair.
