# Supplementary material for: Genome-scale analysis and comparison of gene expression profiles in developing and germinated pollen in Oryza sativa
Source: BMC Genomics. 2010 May 28;11:338. doi: 10.1186/1471-2164-11-338 (PMC2895629; doi:10.1186/1471-2164-11-338)
Supplement: Additional file 6 — Gene Ontology (GO) term "enrichment status" for the pollen stage down-regulated genes in MPGs and GPGs. [file 1471-2164-11-338-S6.PPT]

## Slide 1
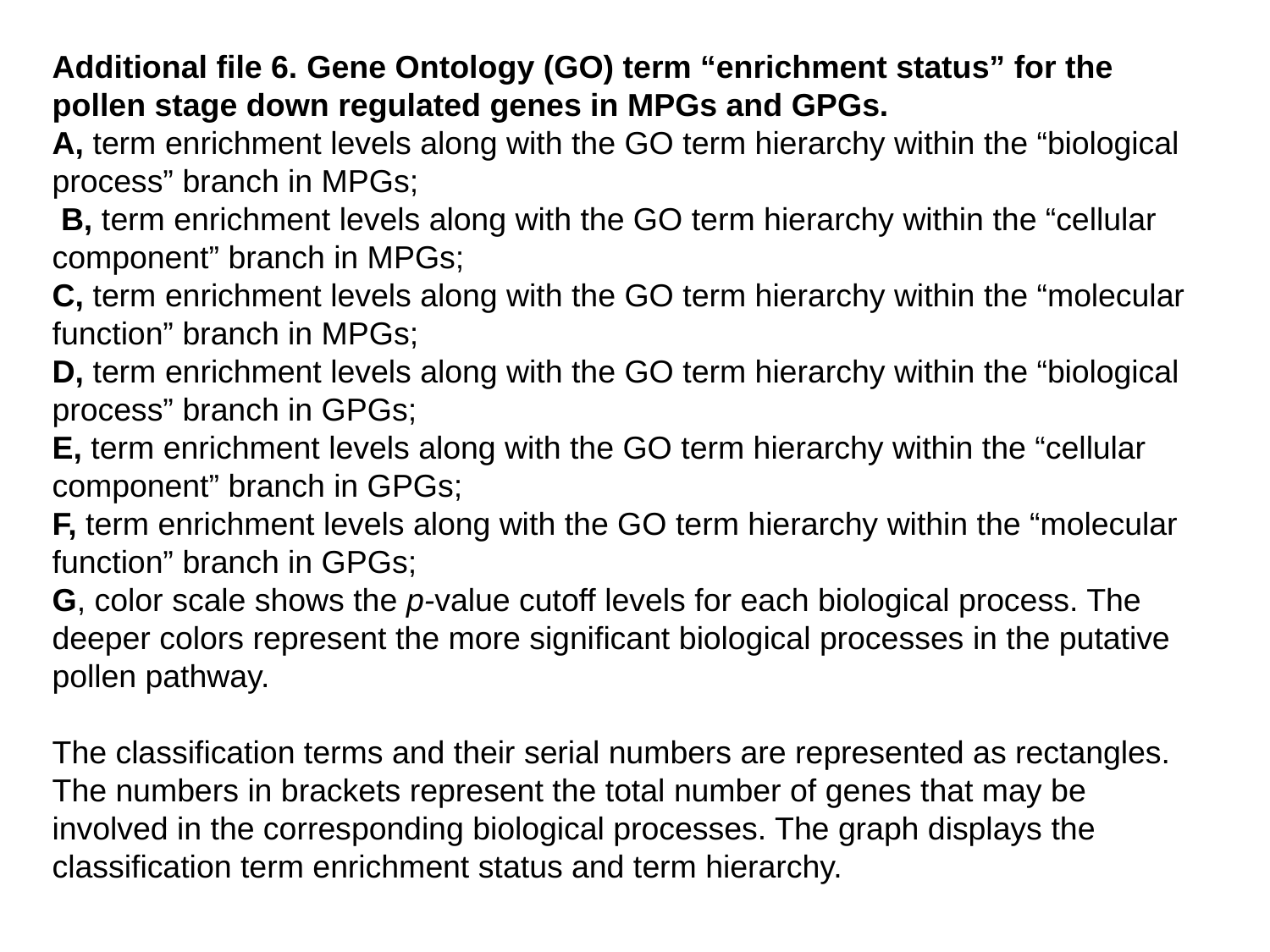

Additional file 6. Gene Ontology (GO) term “enrichment status” for the pollen stage down regulated genes in MPGs and GPGs.
A, term enrichment levels along with the GO term hierarchy within the “biological process” branch in MPGs;
 B, term enrichment levels along with the GO term hierarchy within the “cellular component” branch in MPGs;
C, term enrichment levels along with the GO term hierarchy within the “molecular function” branch in MPGs;
D, term enrichment levels along with the GO term hierarchy within the “biological process” branch in GPGs;
E, term enrichment levels along with the GO term hierarchy within the “cellular component” branch in GPGs;
F, term enrichment levels along with the GO term hierarchy within the “molecular function” branch in GPGs;
G, color scale shows the p-value cutoff levels for each biological process. The deeper colors represent the more significant biological processes in the putative pollen pathway.
The classification terms and their serial numbers are represented as rectangles. The numbers in brackets represent the total number of genes that may be involved in the corresponding biological processes. The graph displays the classification term enrichment status and term hierarchy.

## Slide 2
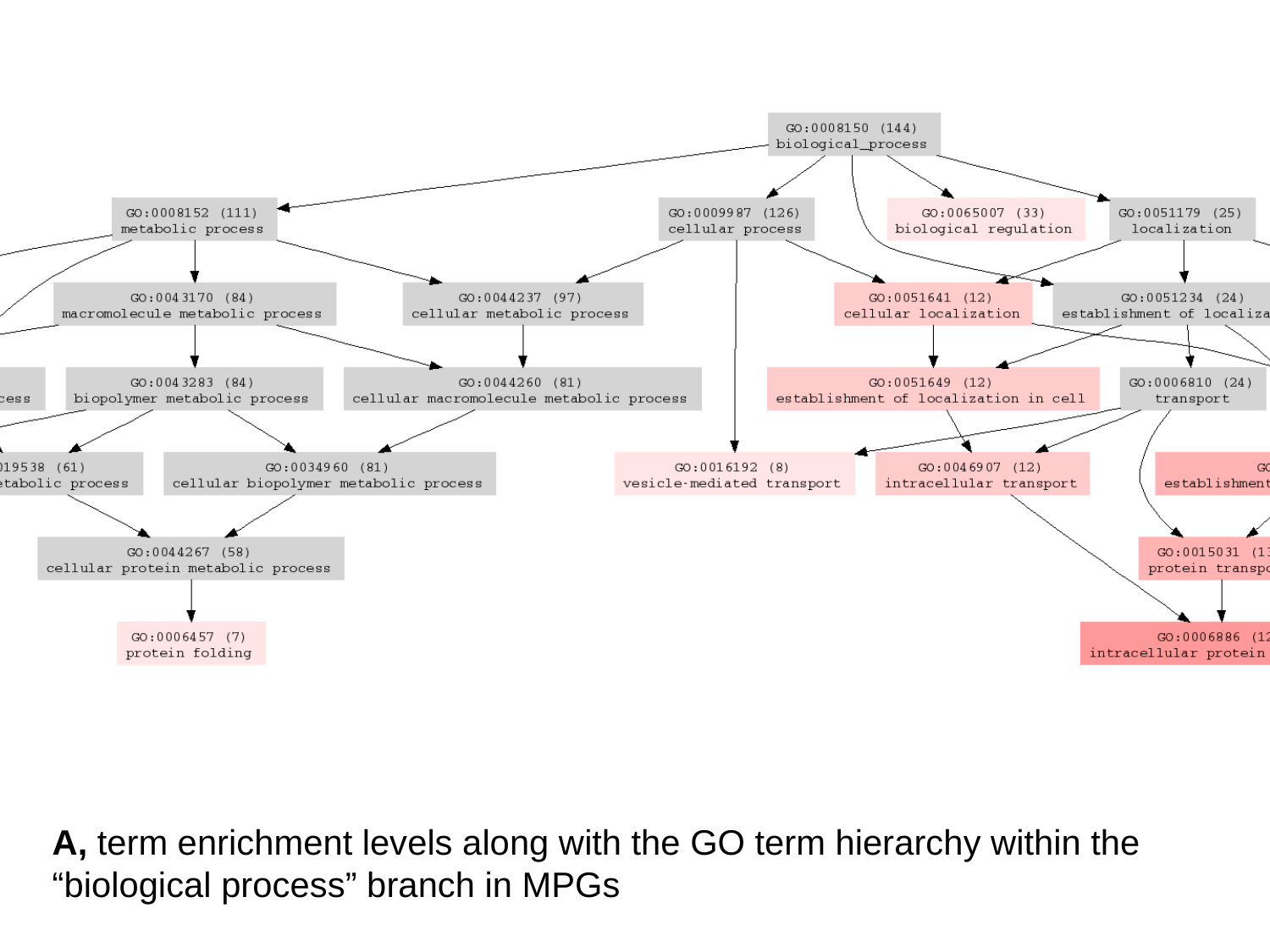

A, term enrichment levels along with the GO term hierarchy within the “biological process” branch in MPGs

## Slide 3
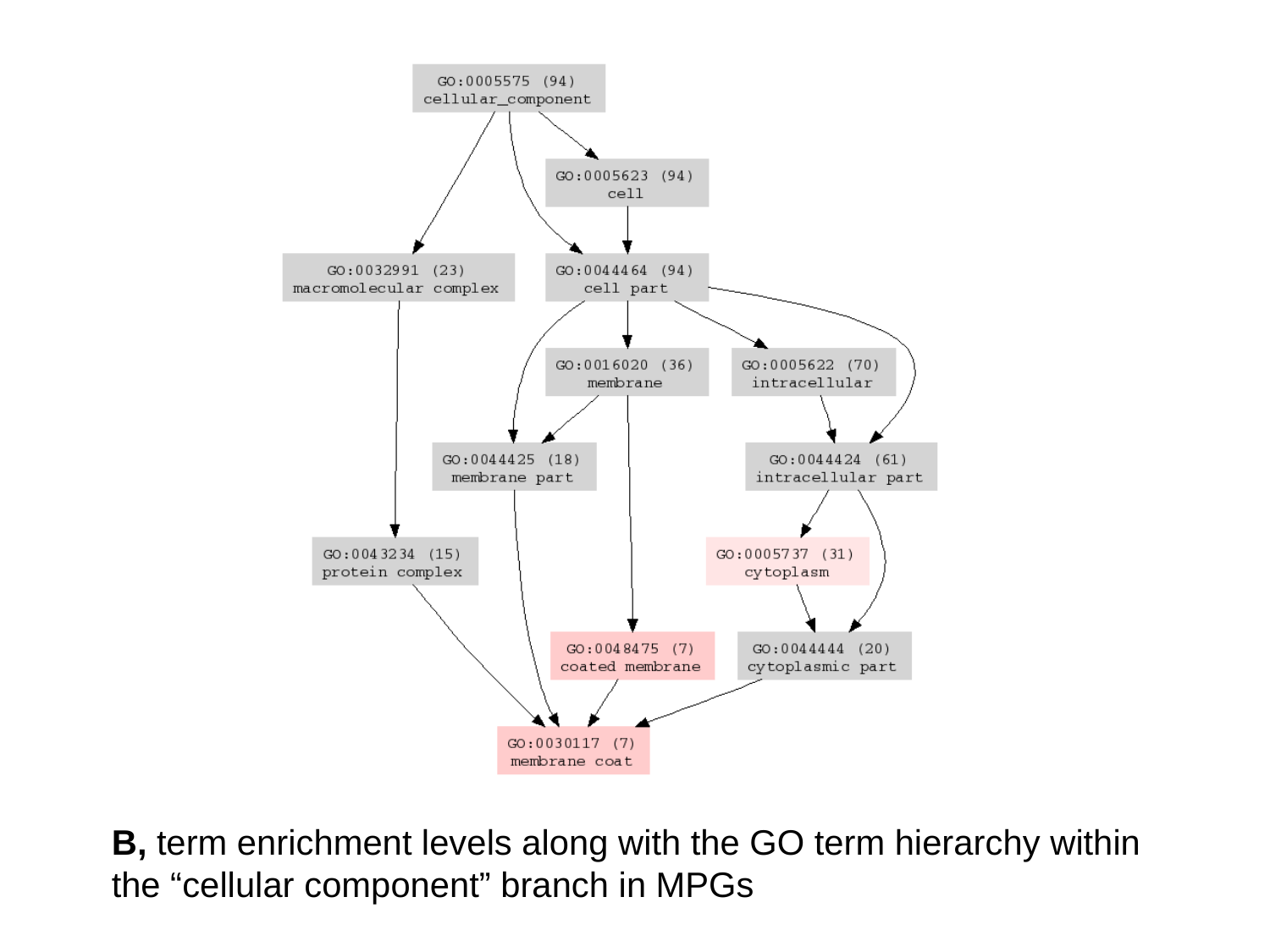

B, term enrichment levels along with the GO term hierarchy within the “cellular component” branch in MPGs

## Slide 4
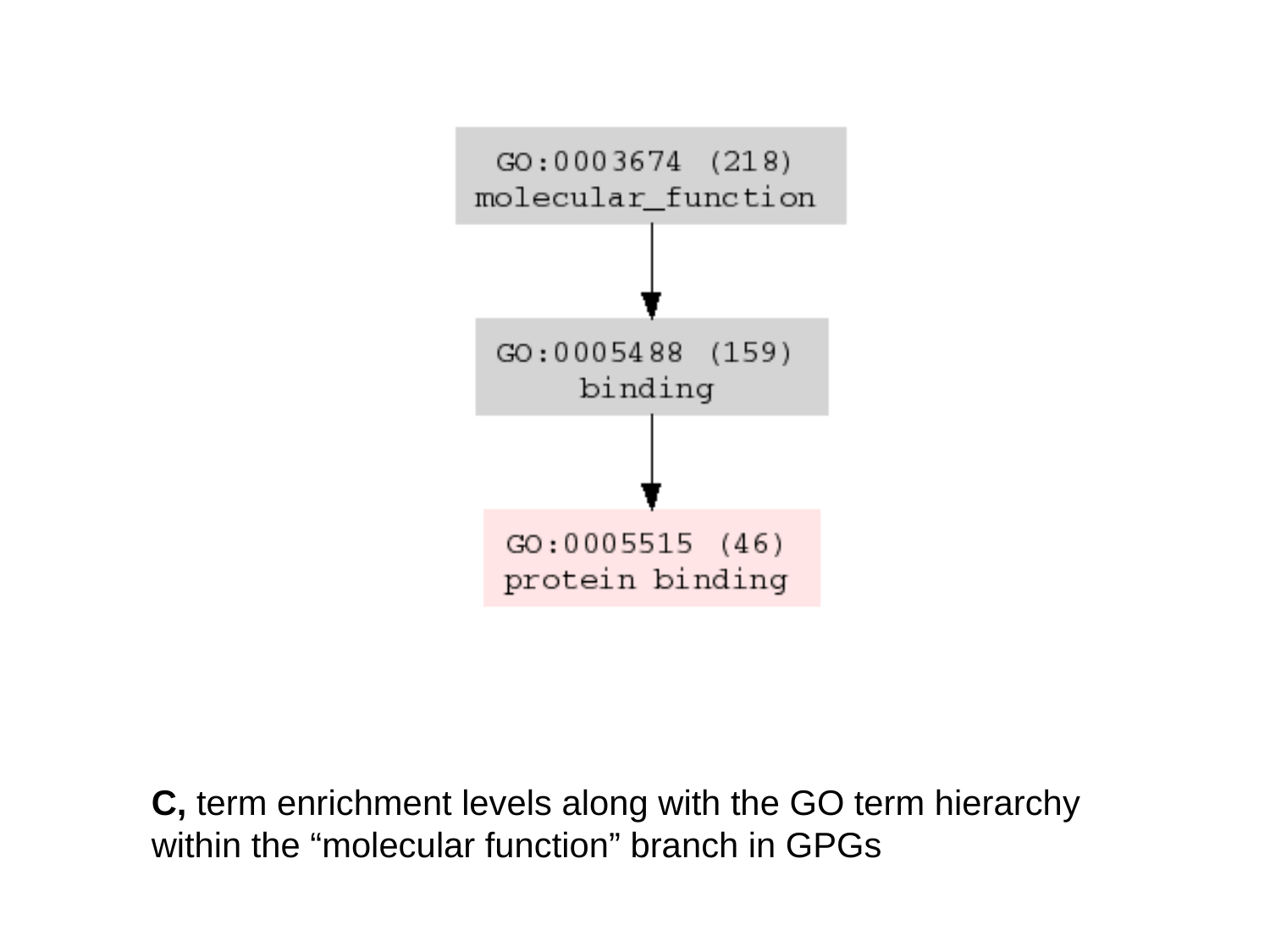

C, term enrichment levels along with the GO term hierarchy within the “molecular function” branch in GPGs

## Slide 5
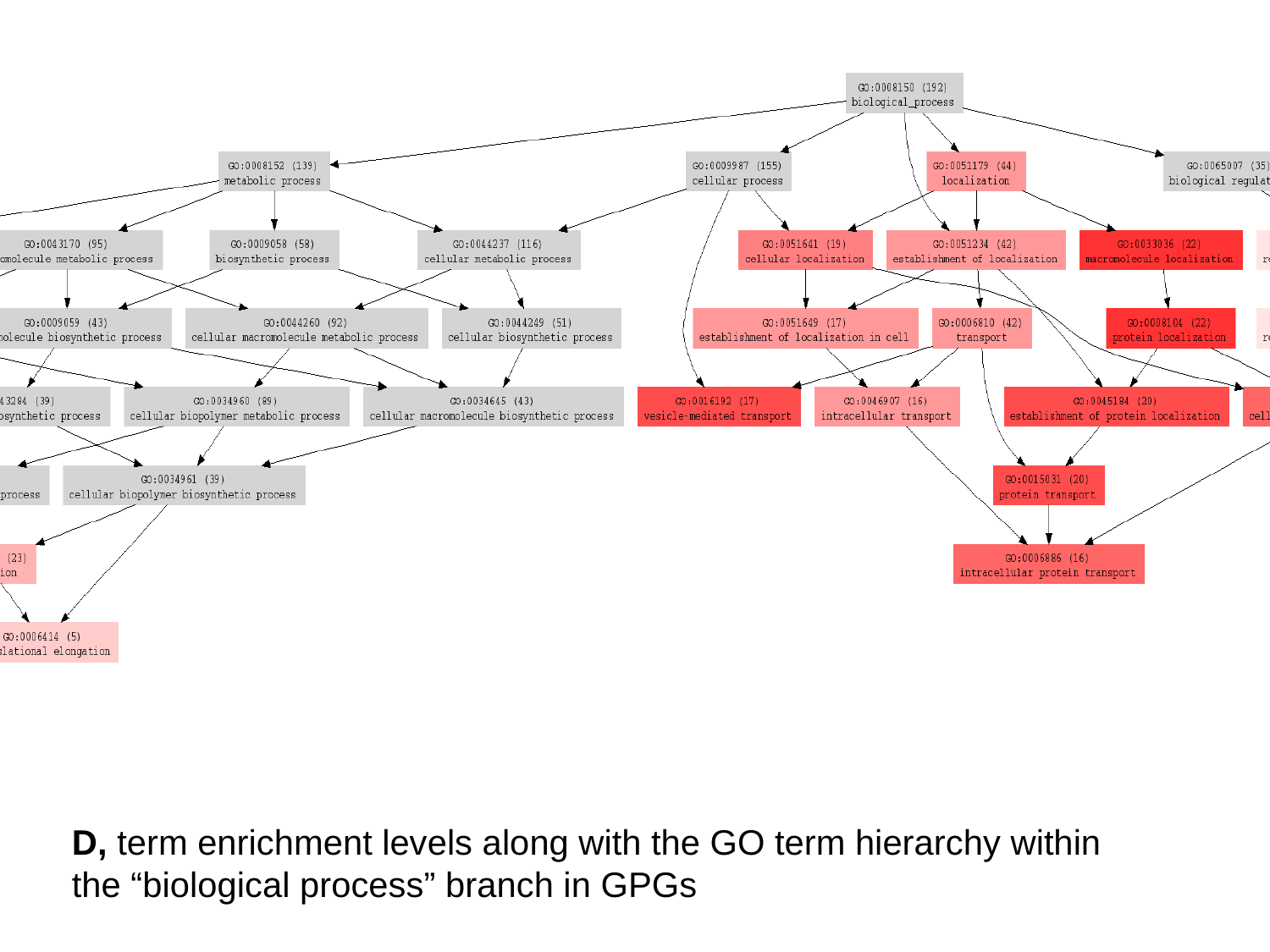

D, term enrichment levels along with the GO term hierarchy within the “biological process” branch in GPGs

## Slide 6
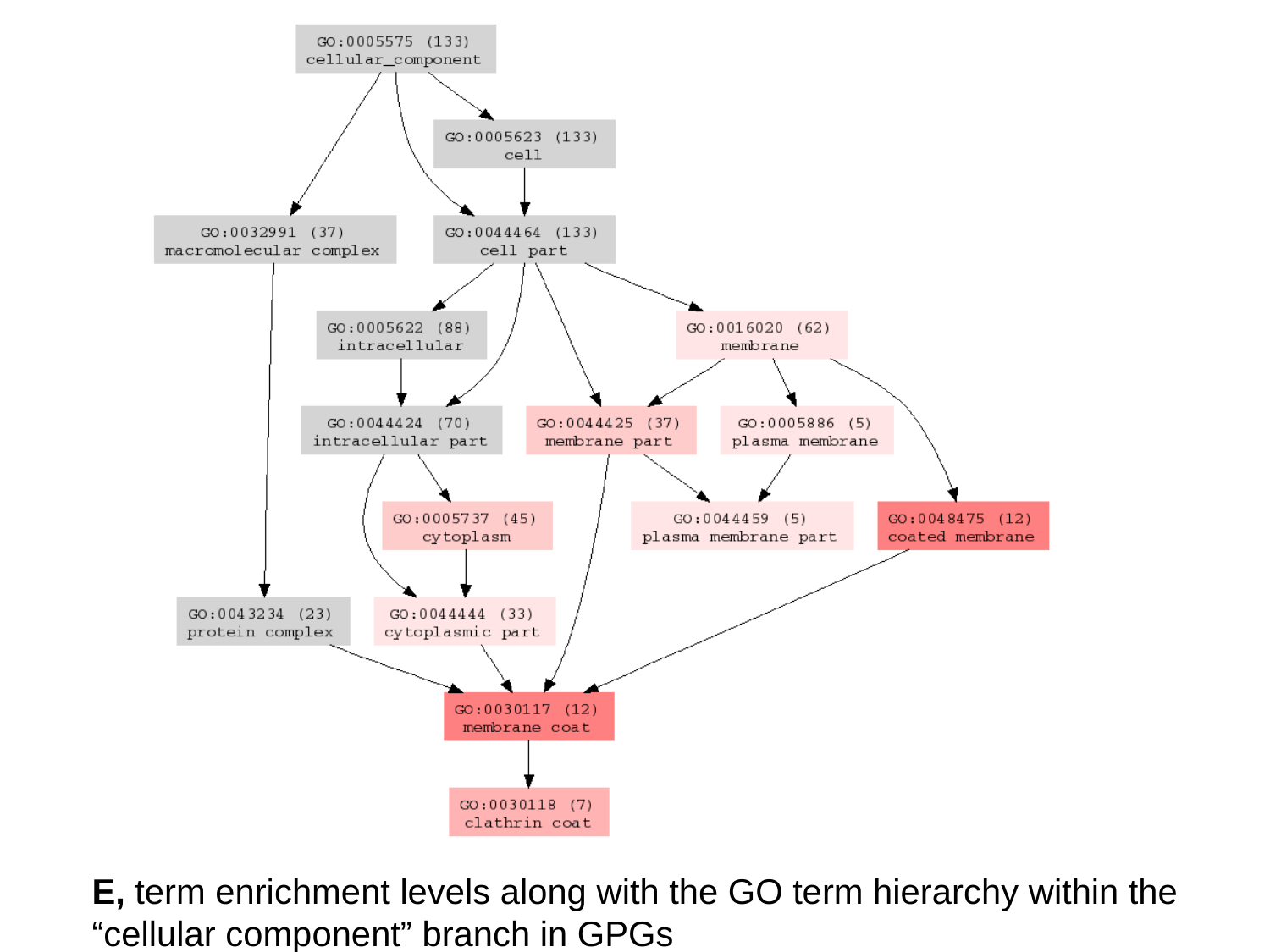

E, term enrichment levels along with the GO term hierarchy within the “cellular component” branch in GPGs

## Slide 7
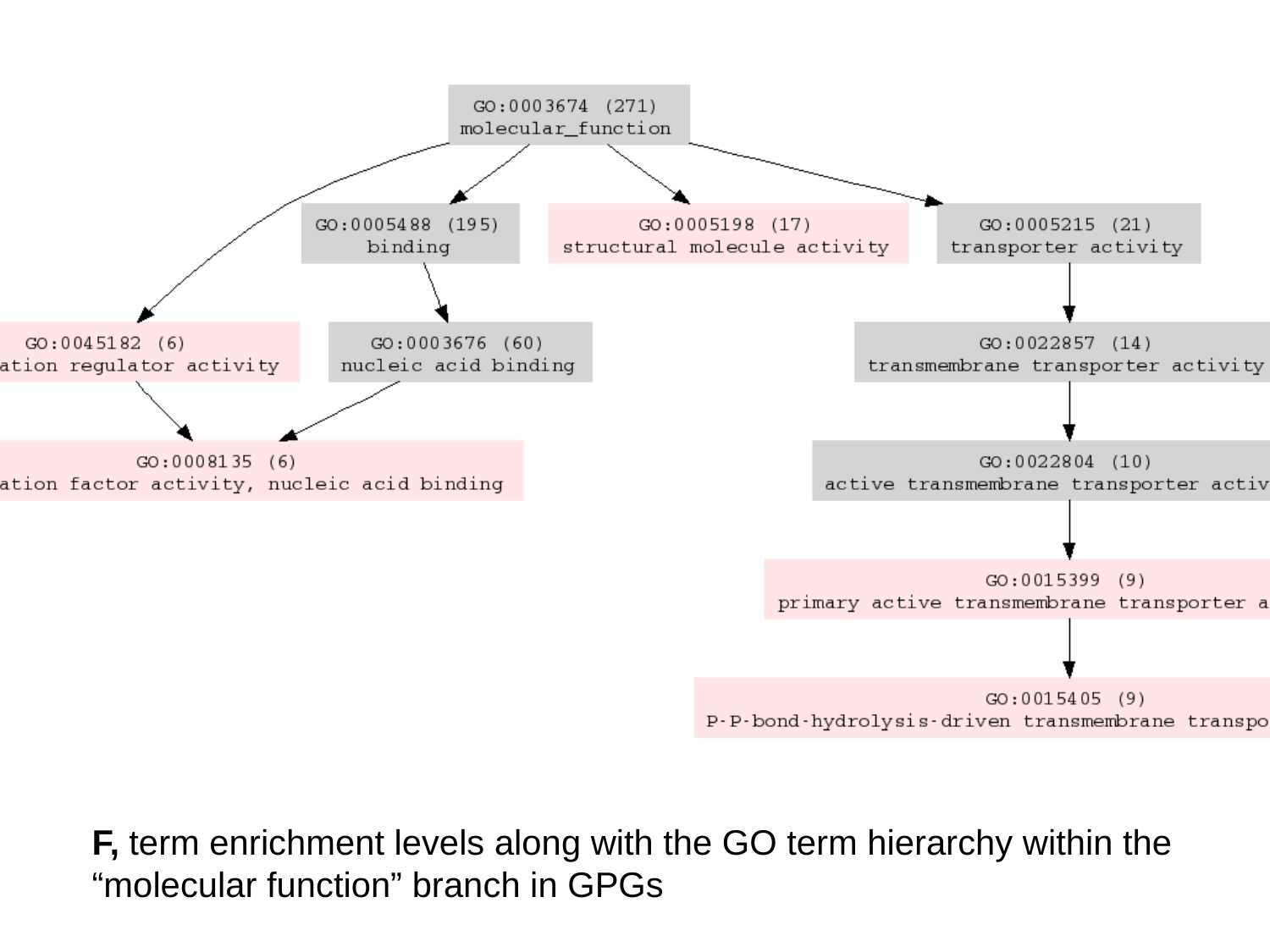

F, term enrichment levels along with the GO term hierarchy within the “molecular function” branch in GPGs

## Slide 8
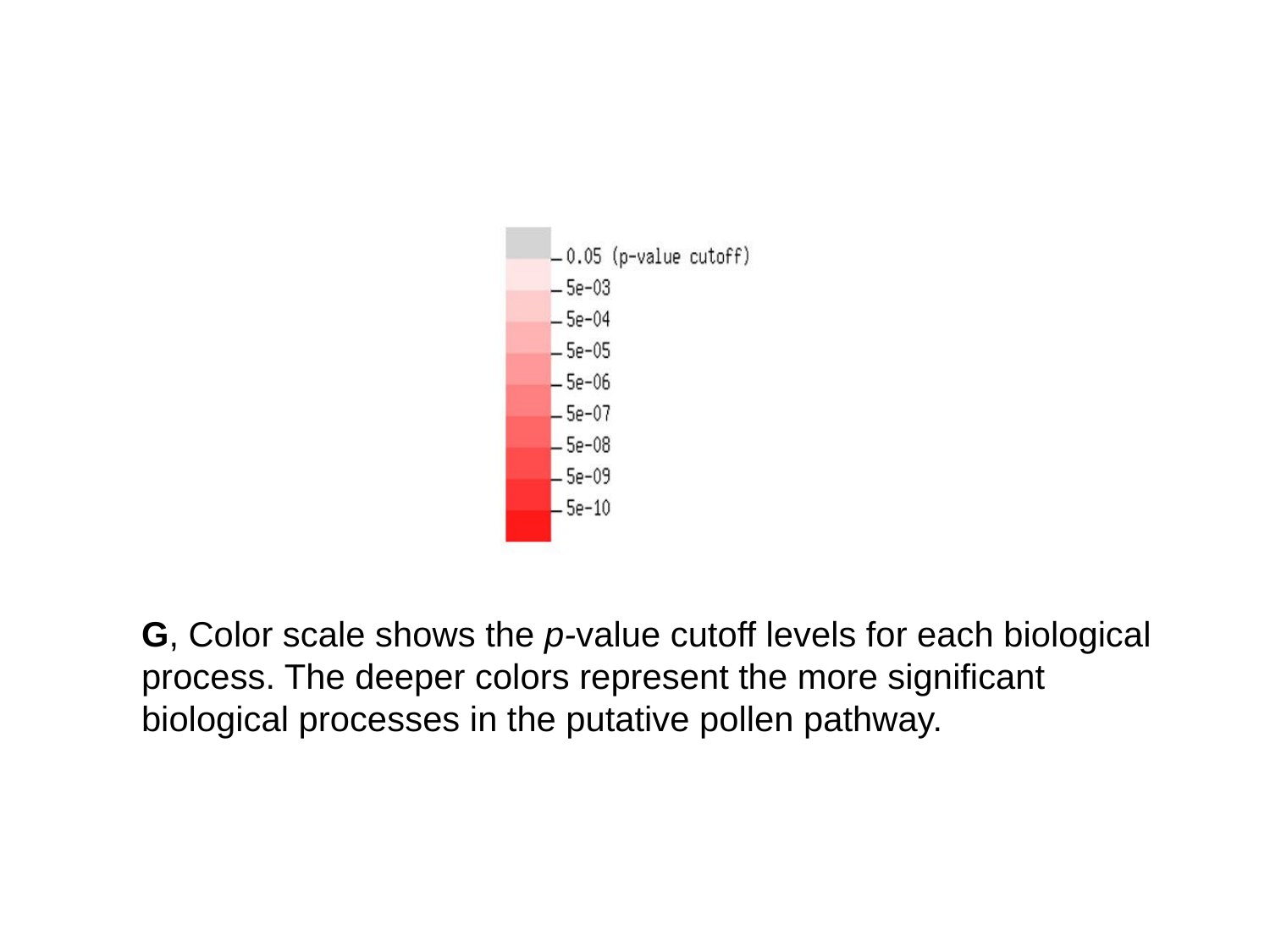

G, Color scale shows the p-value cutoff levels for each biological process. The deeper colors represent the more significant biological processes in the putative pollen pathway.
