# Supplementary material for: Genome-scale analysis and comparison of gene expression profiles in developing and germinated pollen in Oryza sativa
Source: BMC Genomics. 2010 May 28;11:338. doi: 10.1186/1471-2164-11-338 (PMC2895629; doi:10.1186/1471-2164-11-338)
Supplement: Additional file 11 — Comparison of functional groups of stage-enriched genes during pollen development between rice and Arabidopsis. [file 1471-2164-11-338-S11.DOC]

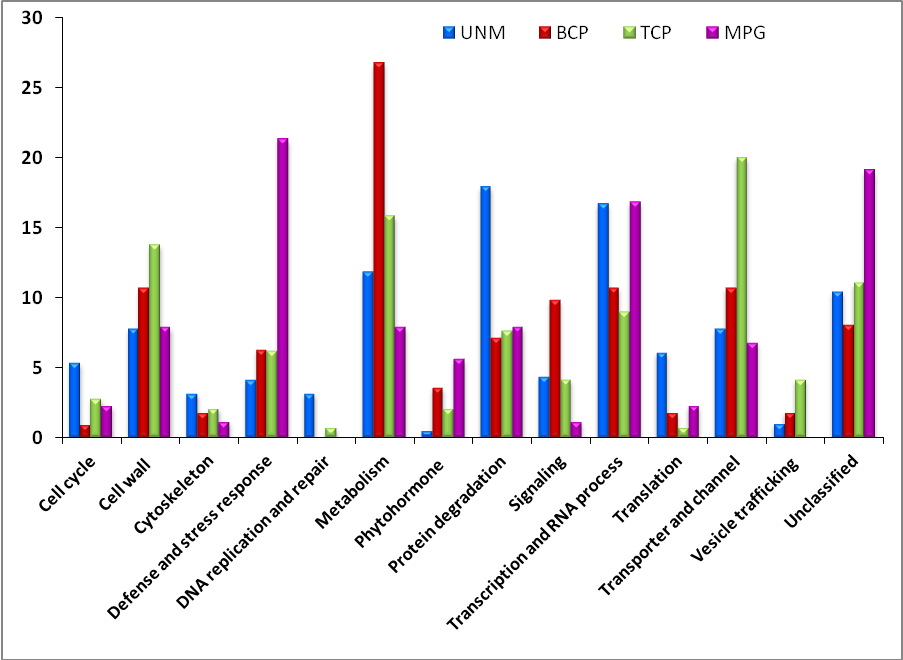


Rice

*Arabidopsis*


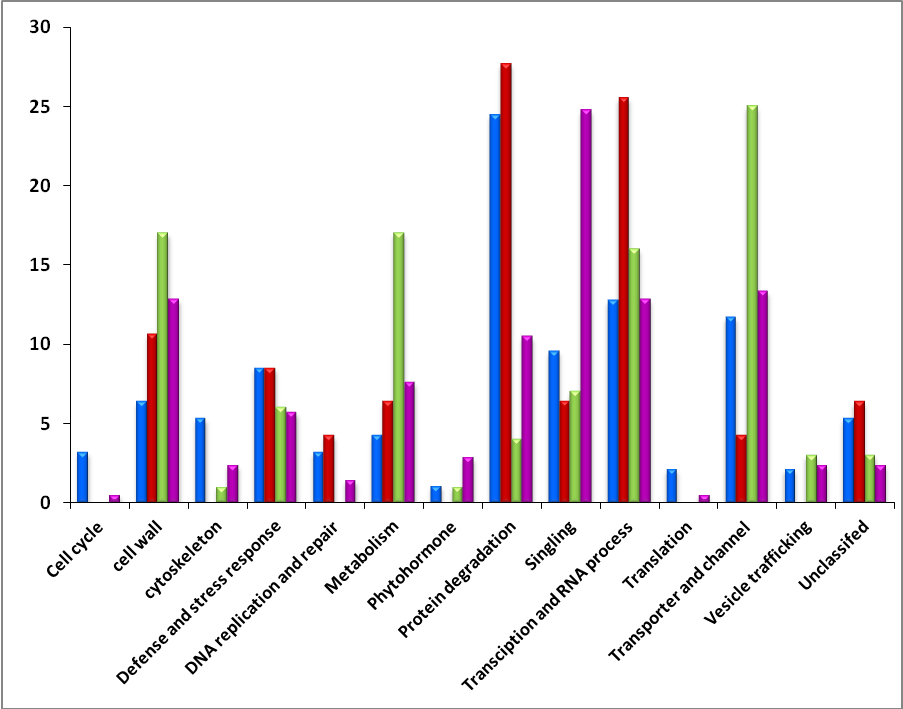


**Additional file 11. Comparison of functional groups of stage-enriched genes during pollen development between rice and A*rabidopsis*.**

The columns demonstrate the percentage of each group in the respective stage. Corresponding detailed information is listed in Additional file 5a and b for rice, and Additional file 12 for *Arabidopsis*.
